# Circulating cell populations as response predictors and targets to improve immunotherapy in metastatic lung cancer

**DOI:** 10.1016/j.isci.2026.116124

**Published:** 2026-06-08

**Authors:** Amanda B. Figueiredo, Guilherme F.B. Evangelista, Stephanie M.I. Ferreira, Larissa M. Kuil, Gabriela Barbeta, Lukas C. Iohan, Andrea T. Faccio, Karina H.M. Cardozo, Valdemir M. Carvalho, Clara M. Cavalcanti, Robert Balderas, Rodrigo P. Lopes, James Turner, Thaiany G. Souza-Silva, Juan C.S. Silva, Jonathan P. Avila, Helder T.I. Nakaya, Nayane A.L. Galdino, Ananda D. Lopes, Kátia L.P. Morais, Iasmim P. Santos, Helano C. Freitas, Jefferson L. Gross, Clóvis A.L. Pinto, Rubens Chojniak, Walderez O. Dutra, Vladmir C. Cordeiro-de-Lima, Kenneth J. Gollob

**Affiliations:** 1 Translational Immuno-oncology Group, International Research Center, A. C. Camargo Cancer Center, São Paulo, SP, Brazil; 2 Translational Immuno-oncology Laboratory, Hospital Israelita Albert Einstein, São Paulo, SP, Brazil; 3 Center for Research in Immuno-oncology (CRIO), Hospital Israelita Albert Einstein, São Paulo, SP, Brazil; 4 Grupo Fleury, São Paulo, SP, Brazil; 5 BD Biosciences, San Jose, CA, USA; 6 School of Sport, Exercise and Rehabilitation Sciences, University of Birmingham, Birmingham, UK; 7 Department of Immunology, Harvard Medical School, Boston, MA, USA; 8 Department of Clinical and Toxicological Analyses, School of Pharmaceutical Sciences, University of São Paulo, São Paulo, SP, Brazil; 9 Computational Systems Biology Laboratory, Education and Research Center, Hospital Israelita Albert Einstein, São Paulo, SP, Brazil; 10 Lung and Thorax Reference Center, A. C. Camargo Cancer Center, São Paulo, SP, Brazil; 11 Pathologic Anatomy Department, A. C. Camargo Cancer Center, São Paulo, SP, Brazil; 12 Diagnostic Imaging Department, A. C. Camargo Cancer Center, São Paulo, SP, Brazil; 13 Department of Morphology, Federal University of Minas Gerais, Belo Horizonte, MG, Brazil

**Keywords:** oncology, therapeutics, immune system

## Abstract

Immunotherapy efficacy varies among patients with advanced non-small cell lung cancer (NSCLC). Here, we profiled baseline systemic immunity in patients with stage IV NSCLC treated with anti-PD-1 plus chemotherapy to identify immune mechanisms and circulating biomarkers associated with response. We prospectively enrolled 33 treatment-naive patients, including 22 responders and 11 non-responders at week 9, and 17 responders and 16 non-responders at 6 months. Responders showed increased frequencies of circulating T cells expressing CD69, TCF-1, and CXCR3. In contrast, non-responders exhibited higher frequencies of CTLA-4-, CD161-, and IL-10-expressing CD4^+^ and CD8^+^ T cells. These systemic immune profiles were mirrored in the tumor microenvironment in an independent cohort. Importantly, combined CTLA-4 and PD-1 blockade reactivated anti-tumor T cell features in non-responders *in vitro*, highlighting CTLA-4 as a potential driver of resistance. Together, these findings support personalized immunotherapy strategies guided by systemic immune biomarkers in advanced NSCLC.

## Highlights

• Baseline blood immune profiles predicted response to immunotherapy with chemotherapy
• Activated circulating T cells were enriched in patients with better clinical outcomes
• Blood and tumor immune profiles showed concordant response-associated T cell states
• Combined ICI blockade restored anti-tumor T cell features in non-responder samples *in vitro*

## Graphical abstract

**Figure:**
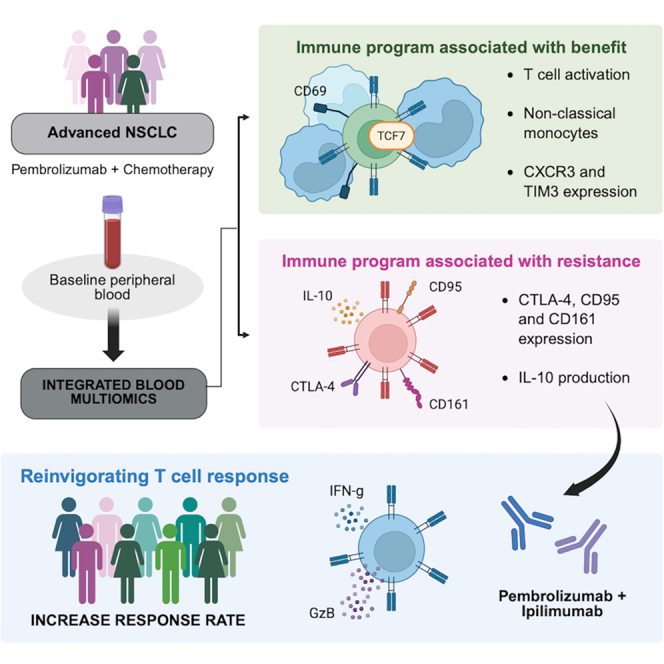

## Introduction

Over the past decade, cancer treatment has evolved significantly beyond the traditional approaches of chemotherapy, radiotherapy, and surgery.^1^ A key advancement has been the development of immunotherapy, particularly with the emergence of immune checkpoint inhibitors such as anti-PD-1 antibodies,^2^ leading to new therapies aimed at reinvigorating an inhibited anti-tumor T cell response.^3^

The use of anti-PD-1 therapy as a first-line treatment of metastatic non-small cell lung cancer (NSCLC) has shown promising results, with studies showing an overall response rate up to 45%.^4^^,^^5^^,^^6^^,^^7^ Furthermore, the KEYNOTE-189 trial demonstrated that patients with NSCLC receiving a combination of pembrolizumab (anti-PD1) and chemotherapy had a higher overall response rate (48.3% vs. 19.9%) and a longer duration of response (12.7 months vs. 7.1 months) compared to chemotherapy alone.^8^ While these therapies have shown success, some patients either do not respond or experience disease progression shortly after an initial response, underscoring the necessity to elucidate the mechanisms that determine therapeutic success or failure. Studies analyzing systemic circulating immune cell subpopulations in patients undergoing treatment with checkpoint inhibitors can provide critical insights into the cellular mechanisms at play and identify potential predictive biomarkers for patient stratification.

In the context of NSCLC, a diverse set of T lymphocyte subpopulations, including CD4^+^ T cells, CD8^+^ T cells, and non-classical T cells,^9^^,^^10^ have been identified as key players in the anti-tumor immune response. In addition, natural killer (NK) cells^11^ and various antigen-presenting cells (APCs) such as monocytes, macrophages, and dendritic cells are also critical.^12^ Dysregulation of the balance between these subpopulations with distinct activation states and suppression mechanisms greatly influences whether a patient will respond or not to immune checkpoint inhibitors. Tumor cells can also alter metabolic pathways to create an environment conducive to their own growth while simultaneously suppressing immune system activation.^13^ This involves the production of metabolites such as adenosine, kynurenine, and prostaglandin E2, not only by the tumor cells themselves but also by regulatory T cells (Treg) and tumor-associated macrophages (TAMs), further reinforcing immune suppression. Lastly, the competition between tumor cells and immune cells for crucial amino acids such as arginine, tryptophan, and glutamine is another mechanism through which tumors can impair T lymphocyte function.^14^^,^^15^

Here, we analyzed the pretreatment systemic immunological status of 33 patients with treatment-naïve stage IV NSCLC undergoing anti-PD1 therapy combined with chemotherapy. Patients were classified as responders or non-responders at either 9 weeks or 6 months post-therapy initiation. The analysis of peripheral blood immune subpopulations provided a snapshot of immune cells likely activated in draining lymph nodes and en route to the tumor, offering a non-invasive source of predictive markers for therapeutic response. Thus, this approach offers valuable insights into the activation states and functional potential of these cells, which are directly relevant to clinical outcomes.

To investigate the immune mechanisms associated with treatment outcome, we analyzed the soluble and cellular immune profiles in peripheral blood using multiplex bead assay, high-parameter flow cytometry, and scRNAseq, along with plasma metabolomics using UPLC-mass spectrometry. We further investigated similar T cell subpopulations found in the blood within the TME using single-cell RNAseq data from a publicly available independent patient cohort. Our findings elucidate mechanisms of therapeutic failure and identify potential markers of response, providing valuable insights for improving immunotherapy strategies in stage IV NSCLC.

## Results

### Patient response characteristics

We enrolled 33 patients with stage IV NSCLC in this prospective study and observed a 42.5% objective response rate. Although 33 patients were enrolled, baseline whole blood immune profiling was available for 27 individuals, metabolomics profiling was available for 32 patients, baseline high-dimensional PBMC immune profiling was available for 25 patients, and scRNA-seq data were available for 12 patients, due to sample availability and/or quality control criteria. Clinical outcomes were assessed for all enrolled participants. All figure legends indicate the number of samples analyzed for each panel. Analysis showed no significant correlation between response and factors such as sex, metastasis site, driver mutations, PD-L1 expression, histology, or chemotherapy regimen (Table S1). After 9 weeks of beginning the treatment, patients were classified into responders and non-responders. Patients with disease progression were included in the “non-responder” group, and patients with partial response or stable disease were included in the “responder” group. Notably, responders were older (median age 69 vs. 62.5 years in non-responders, *p* = 0.0492). Non-responders had a shorter median follow-up (7.5 vs. 20.2 months, *p* = 0.0117) and lower overall survival (7.8 vs. 14.3 months, *p* = 0.0227, hazard ratio = 2.206) as compared to responders. Machine learning analysis identified progression-free survival (PFS), follow-up time, and age as key clinical data features (Figure S1A). A Random Forest Classifier (RFC) model using the clinical data achieved 90% accuracy in predicting response status, as shown in a PCA plot (Figure S1B).

### Circulating cell populations segregate patients with NSCLC by therapeutic response

**Figure 1:**
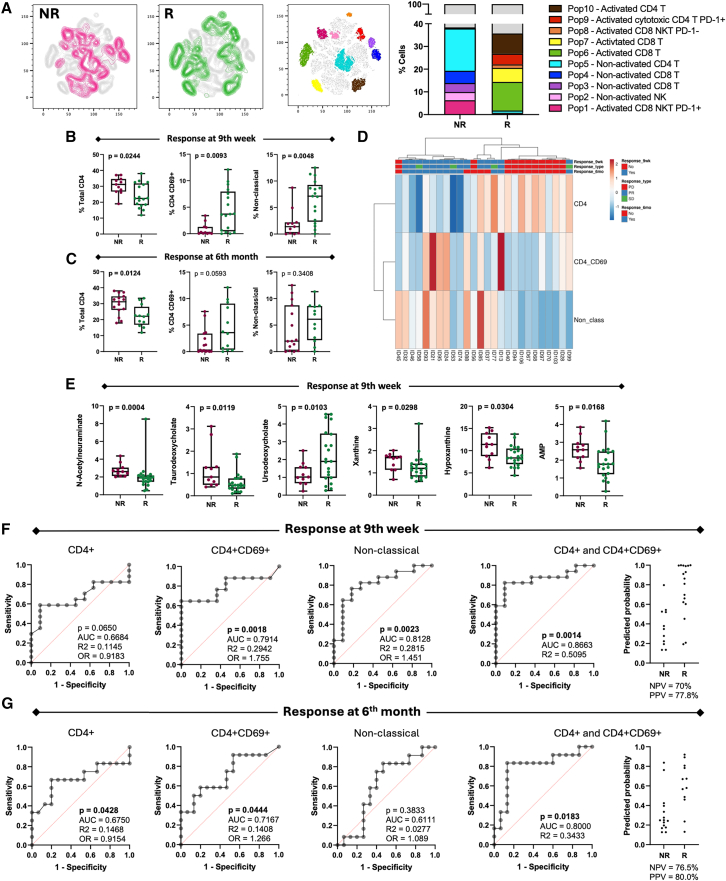
Distinct systemic cellular and metabolomic profiles distinguish responder and non-responder patients with NSCLC treated with pembrolizumab (anti-PD1) and chemotherapy Peripheral blood samples from patients with NSCLC were collected before treatment initiation. After 9 weeks or 6 months from the start of the treatment, clinical response was evaluated, and patients were identified as either non-responders (NR, pink symbols) or responders (R, green symbols). Median and interquartile range are shown. (A) tSNE for NK and T lymphocyte populations. Ten lymphocyte populations differentially present in the two groups of patients were selected and analyzed for the expression of CD3, CD4, CD8, CD56, PD-1, CD69, and CD107a (*n* = 3 NR, *n* = 3 R). (B) Frequency of CD4^+^ T lymphocytes, CD4^+^CD69^+^ T lymphocytes, and non-classical monocytes in peripheral blood of patients with NSCLC segregated into responder and non-responder patients at the 9^th^ week of treatment (*n* = 11 NR, *n* = 16 R). (C) Frequency of CD4^+^ T lymphocytes, CD4^+^CD69^+^ T lymphocytes, and non-classical monocytes in peripheral blood of patients with NSCLC segregated into responder and non-responder patients at the 6^th^ month of treatment (*n* = 15 NR, *n* = 12 R). (D) Unsupervised hierarchical cluster graph and heatmap of responders and non-responders using CD4^+^ T lymphocytes and non-classical monocyte data. (E) Relative comparison of plasma analytes using ultra-performance liquid chromatography and high-resolution mass spectrometry compared between non-responder and responder groups (*n* = 11 NR, *n* = 21 R). (B, C, and E) Statistical analysis using Mann-Whitney or Student’s t tests according to Gaussian distribution. The accuracy of immune cell populations to predict response was assessed using ROC curves. AUC, *p* value, Cox-Snell’s R squared and odds ratio are shown after simple logistic regression analysis (1 parameter) and AUC, *p* value, Nagelkerke’s R squared, and negative (NPV) or positive (PPV) predictive values are shown after multiple logistic regression analysis (2 parameters). (F) The accuracy of CD4^+^ T lymphocytes, CD4^+^CD69^+^ T lymphocytes, and non-classical monocytes to predict the response of patients with NSCLC at the 9^th^ week of treatment (*n* = 11 NR, *n* = 16 R). (G) The accuracy of CD4^+^ T lymphocytes, CD4^+^CD69^+^ T lymphocytes, and non-classical monocytes to predict the response of patients with NSCLC at the 6^th^ month of treatment (*n* = 15 NR, *n* = 12 R).

Using flow cytometry on whole blood samples, we conducted analyses to discern immune cell populations linked to responder or non-responder status as determined at 9 weeks following treatment initiation in patients with NSCLC. Initially, we applied tSNE, an unsupervised algorithm, to flow cytometry data. Our findings (Figure 1A) indicate distinct cell populations present pre-treatment that are associated with either the responder or non-responder groups. Further analysis based on specific markers (CD3, CD4, CD8, CD56, PD-1, CD69, and CD107a) showed that activated cells (CD69^+^ or CD107a+) were predominant in responders. In contrast, non-responders had a higher frequency of non-activated cells, with the exception of a subset of CD8 NKT cells expressing PD-1, indicative of a potentially exhausted phenotype (Figure 1A).

In a targeted analysis of the flow cytometry data using all samples, we confirmed that responders had a significantly higher frequency of activated CD4^+^CD69^+^ T lymphocytes and a lower frequency of total CD4^+^ T lymphocytes (Figure 1B), supporting our t-SNE analysis results. No significant differences were found in CD8^+^ T lymphocyte subpopulations. Importantly, total CD4^+^ T cells were quantified as a proportion of total T lymphocytes (CD3^+^), reflecting CD4^+^/CD8^+^ distribution rather than absolute counts. Consistently, the total number of PBMCs recovered at baseline, normalized for the volume of peripheral blood processed, did not differ between responders and non-responders (*p* = 0.584), supporting that these differences are not associated with overall leukocyte cellularity. Further analysis of blood-derived monocyte subpopulations, classified by CD14 and CD16 expression, showed that non-classical CD14^−^CD16^+^ monocytes were more prevalent in the responder group as determined at 9 weeks post-treatment initiation (Figure 1B). Other cell populations were analyzed, including double-negative (DN) T lymphocytes, NK cells, and B lymphocytes, but we found no significant differences between the two groups, responder and non-responder patients (data not shown).

In addition to the 9-week response evaluation, we also analyzed the initial immune profile with respect to durable responses at 6 months post-treatment. We examined 17 non-responders, including 6 initially classified as responders at 9 weeks, and 16 consistent responders. This analysis reaffirmed our earlier findings: Patients with durable responses showed a higher frequency of activated CD4^+^CD69^+^ T lymphocytes pre-treatment than non-responders. Notably, total CD4^+^ T lymphocyte frequency was elevated in non-responders. Unlike the 9-week assessment, however, we found no significant difference in non-classical monocyte frequency between the groups at this stage (Figure 1C).

We conducted an unsupervised cluster analysis using the three cell subpopulations (CD4^+^ T lymphocytes, CD4^+^CD69^+^ T lymphocytes, and non-classical monocytes) that showed differential expression between responders and non-responders. This analysis effectively segregated the two groups (Figure 1D). The resulting heatmap displayed a non-responder cluster characterized by high CD4^+^ T cell frequency but low activated CD4^+^CD69^+^ T cell and non-classical monocyte frequencies, while the responder cluster showed the reverse pattern. These findings suggest that these three cell subpopulations could be explored as promising biomarkers for predicting immunotherapy response in patients with NSCLC.

To further unravel potential immunoregulatory networks correlating with immunotherapeutic outcomes in NSCLC, we conducted an analysis of 48 soluble immune factors in patient plasma encompassing a range of inflammatory and anti-inflammatory cytokines, chemokines, and growth factors. Despite this comprehensive approach, our findings revealed no significant differences in the plasma concentrations of these factors between responders and non-responders (data not shown).

In addition, we explored the relationship between specific immunoregulatory plasma metabolites in patients with NSCLC and their response to immunotherapy, assessed at 9 weeks post-treatment (Table S2). Intriguingly, metabolites such as N-acetylneuraminate, taurodeoxycholate, xanthine, and AMP were found in higher concentrations in the plasma of non-responders (Figure 1E). This observation is particularly noteworthy, as it suggests a potential metabolic underpinning in the immune response. The lower frequency of activated CD4^+^CD69^+^ T lymphocytes, coupled with increased levels of these down-modulatory metabolites in non-responders, is consistent with an immunosuppressive milieu.

To assess the ability of systemic immune cell populations to predict immunotherapy response in patients with NSCLC, we applied logistic regression and performed ROC curve analysis. Activated CD4^+^CD69^+^ T lymphocytes emerged as a potent early predictor of response at 9 weeks, showing high specificity and sensitivity (Figure 1F). Combining total CD4^+^ T lymphocytes with CD4^+^CD69^+^ T lymphocytes enhanced this predictive capability, resulting in an area under the curve (AUC) of 0.8663 (R2 = 0.5095), and a positive predictive value of 77.8%, with a negative predictive value of 70%. Additionally, higher frequencies of non-classical monocytes in blood emerged as a significant early response indicator.

For predicting durable response at 6 months, CD4^+^CD69^+^ T lymphocytes maintained their predictive reliability (Figure 1G), with an AUC of 0.7167. Incorporating total CD4^+^ T lymphocytes into a multivariable analysis yielded an AUC of 0.8000 (R2 = 0.3433), with positive and negative predictive values of 80% and 76.5%, respectively.

Lastly, we assessed the potential of baseline plasma metabolites in patients with NSCLC to serve as predictors for immunotherapy response. Our findings revealed that taurodeoxycholate, xanthine, and hypoxanthine had significant predictive values for therapeutic failure (Figure S2). Complementing this, a machine-learning feature importance analysis identified lysophosphatidylcholine (LPC-18:2), xanthine, and cyclamic acid as key predictors for identifying responders, achieving an accuracy of 95% (Figures S1E and S1F). Notably, high levels of LPC -18:2 were characteristic of responders. In contrast, increased concentrations of xanthine and cyclamic acid were associated with non-responders. This discovery is particularly intriguing given the recent association of elevated LPC -18:2 levels with the inhibition of lung cancer cell proliferation.^16^

Histology (adenocarcinoma versus squamous cell carcinoma) was not significantly associated with response in this cohort; furthermore, excluding SCC cases (6/33 patients) did not alter the main baseline immune associations observed between responders and non-responders, supporting that the identified signatures are not primarily driven by histological subtype in this cohort.

### CD4 T lymphocytes are key predictors of PFS in NSCLC

In our subsequent investigation, we evaluated whether predictive markers identified through baseline *ex vivo* whole blood analysis were associated with PFS in patients with NSCLC. The median follow-up time for all patients was 16.7 months, with a median PFS of 5.2 months. Using Youden’s index, we categorized patients into groups with high or low frequencies of specific immune cell populations in their blood samples to evaluate their correlation with PFS.

**Figure 2:**
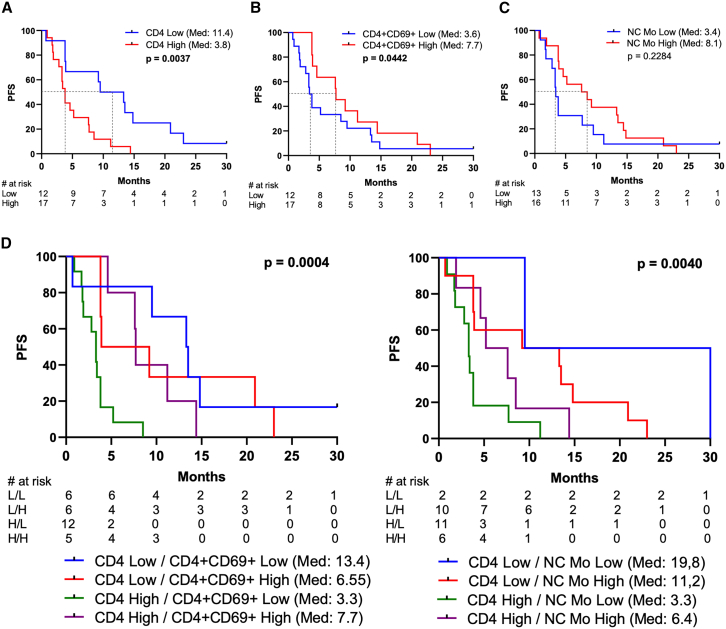
Progression-free survival (PFS) curves of patients with NSCLC treated with pembrolizumab and chemotherapy PFS curves according to the frequency of (A) CD4^+^ T lymphocytes, (B) CD4^+^CD69^+^ T lymphocytes or (C) non-classical monocytes in peripheral blood before starting the treatment. Patients were segregated into Low and High frequency groups according to cutoffs established with Youden’s index (*n* = 11 NR, *n* = 16 R). We show the median survival of the group low (med low) and high (med high). *p* value was determined with the log rank Mantel-Cox test. (D) PFS curves according to combined parameters. Median survival is shown in parentheses.

Our findings indicated that patients with higher frequencies of total CD4^+^ T lymphocytes experienced a shorter median PFS (3.8 months) compared to those with lower frequencies (11.4 months; *p* = 0.0037) (Figure 2A). Conversely, a higher frequency of activated CD4^+^CD69^+^ T lymphocytes was significantly associated with a longer median PFS (7.7 months vs. 3.6 months; *p* = 0.0442) (Figure 2B). However, we observed no significant association between non-classical monocyte frequencies and PFS (Figure 2C).

In a comprehensive survival analysis incorporating all three variables (total CD4^+^ T lymphocytes, activated CD4^+^CD69^+^ T lymphocytes, and non-classical monocytes), the data revealed that a combination of high CD4^+^ T lymphocyte frequency with low frequencies of activated CD4^+^CD69^+^ T lymphocytes or non-classical monocytes correlated with poor PFS, presenting a median PFS of only 3.3 months (Figure 2D).

### CTLA-4 expression associates with a lack of response to immunotherapy

Following the discovery that distinct CD4^+^ T lymphocyte populations were associated with varying responses to immunotherapy in patients with NSCLC, we expanded our investigation into the immune mechanisms underlying therapeutic response. Using high-dimensional flow cytometry panels, we analyzed the functional potential and activation states of unstimulated, *ex vivo*, peripheral blood mononuclear cell (PBMC) derived T lymphocyte and myeloid subpopulations. Importantly, as before, these PBMCs were sampled at baseline, prior to the commencement of therapy. Moving forward, patients will be classified as responders or non-responders based on their evaluation at 9 weeks post-treatment. Using the data obtained, we constructed a heatmap and a volcano plot to visualize cell populations that were distinctively present in either group. Our findings revealed that non-responder patients had an increased presence of cell populations expressing CTLA-4, CD161, CD95, IL-10, or PD-L1/PD-L2. Conversely, responders were characterized by a higher frequency of cell populations expressing the transcription factor TCF-1, as detailed in Figure S3. This difference in cell population profiles between responders and non-responders offers critical insights into the variable immune responses observed in patients with NSCLC undergoing therapy.

**Figure 3:**
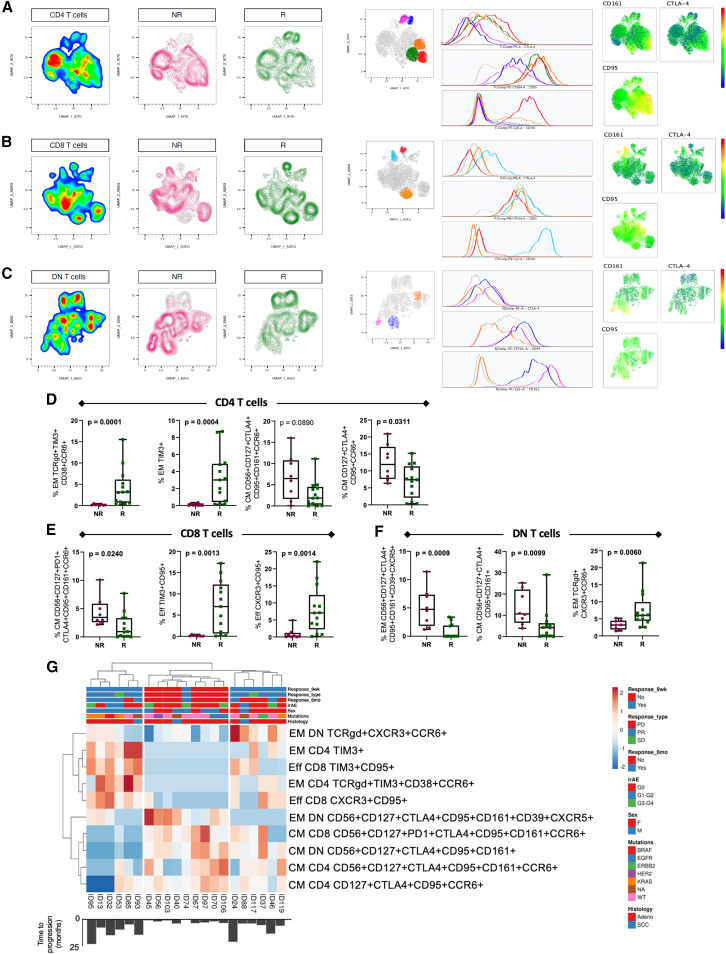
T lymphocytes expressing CTLA-4, CD161, and CD95 are associated with no response to immunotherapy in patients with stage IV NSCLC PBMC from samples collected before starting the treatment were analyzed by high-dimensional flow cytometry, and patients were identified as either non-responders (pink) or responders (green) at the 9^th^ week of treatment. UMAP graphs for (A) CD4, (B) CD8, or (C) double-negative T lymphocyte populations. Ten lymphocyte populations differentially present in the two groups of patients were selected and analyzed for the expression of CTLA-4, CD161, and CD95. (D–F) Frequency of each T lymphocyte population in responder and non-responder patients with NSCLC (*n* = 8 NR, *n* = 13 R). Median and interquartile range are shown. Statistical analysis using Mann-Whitney or Student’s t tests according to the Gaussian distribution. (G) Unsupervised hierarchical cluster graph and heatmap of responder and non-responder patients using differentially expressed subpopulations between the two groups of patients.

Next, we conducted an unsupervised analysis using uniform manifold approximation and projection (UMAP) algorithms to examine subpopulations of CD4^+^ T lymphocytes (Figure 3A), CD8^+^ T lymphocytes (Figure 3B), and DN T lymphocytes (Figure 3C) in each patient group. This analysis aimed to delve deeper into the potential mechanisms associated with therapeutic failure in patients with NSCLC. Our findings indicated that cell populations most frequently found in non-responder patients were characterized by the elevated expression of CTLA-4, CD161, and CD95. We extended our characterization to include an assessment of other exhaustion markers, chemokine receptors, and the memory status of these cells. Interestingly, in responder patients, the predominant cell populations mainly expressed TIM-3 and CXCR3, regardless of their memory status, as depicted in Figures 3D–3F. Lastly, high-dimensional flow cytometry data were explored to identify the immune networks underlying therapeutic response in the patients with NSCLC. The resulting heatmap revealed three distinct clusters of patients, each characterized by a unique pattern of immune cell populations (Figure 3G). Notably, the first cluster demonstrated a durable response (6 months) and a higher frequency of favorable CD4, CD8, and DN T cell effector memory (EM) and effector (Eff) T cell subpopulations expressing TIM-3 and CXCR3. In contrast, the second cluster comprised the non-responsive phenotype and a higher frequency of central memory (CM) T cells expressing inhibitory molecules, including CTLA-4 (in all subpopulations), PD-1, CD95, and CD161, and low levels of the beneficial T cell subsets. Strikingly, the third cluster of patients who initially responded to therapy but later relapsed exhibited a mixed pattern of “good” (i.e., EM and Eff T cells expressing TIM-3 and CXCR3) and “bad” (i.e., CM T cells expressing CTLA-4, PD-1, CD95 and CD161) immune cell populations, suggesting a dynamic interplay between these subsets (Figure 3G). Importantly, these “good” and “bad” phenotypes were also clearly associated with improved or worse months of PFS, respectively (Figure 3G).

### Baseline functional immune states defined by IL-10 and TCF-1 distinguish responders and non-responders

Next, we assessed the functional status of lymphocytes derived from responder and non-responder patients with NSCLC by stimulating cells overnight using a polyclonal or antigen-specific stimulus (overlapping SOX-2 – SRY-box transcription factor 2 – peptides), followed by the examination of immune molecules related to the suppression or activation of immune responses. Again, this analysis was performed at baseline before therapy began.

**Figure 4:**
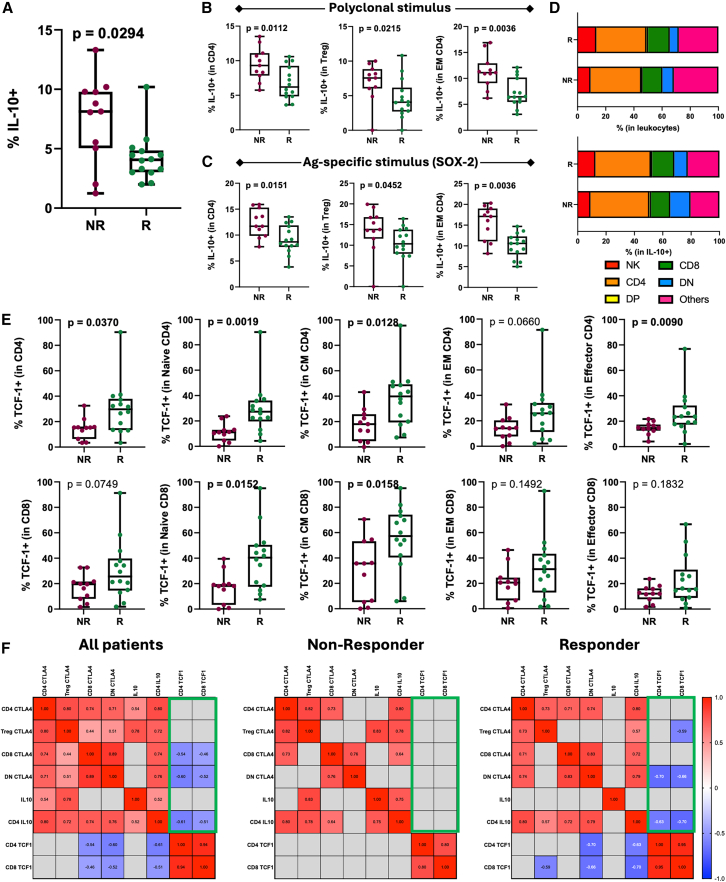
T lymphocytes from responder patients present lower IL-10 expression and higher TCF-1 expression after polyclonal or antigen-specific stimulation PBMC from samples collected before starting the treatment were analyzed by high-dimensional flow cytometry, and patients were identified as either non-responders (pink) or responders (green) at the 9^th^ week of treatment (*n* = 11 NR, *n* = 14 R). (A) IL-10 expression in leukocytes. (B) IL-10 expression in total CD4, regulatory, or effector memory CD4 T lymphocytes after polyclonal stimulus with anti-CD3 and anti-CD28 antibodies. (C) IL-10 expression in total CD4, regulatory or effector memory CD4 T lymphocytes after antigen-specific stimulation with SOX-2 overlapping peptides and anti-CD28 antibody. (D) Frequencies of NK cells and T lymphocytes in leukocytes or IL-10+ cells from non-responder and responder patients. (E) TCF-1 expression in CD4 or CD8 T lymphocyte populations. (A–C and E) Median and interquartile range are shown. Statistical analysis using Mann-Whitney or Student’s t tests according to the Gaussian distribution. (F) Correlation matrices for CTLA-4+, IL-10+, or TCF-1+ T lymphocyte populations in all patients or in non-responder or responder patients. Numbers in the boxes represent Spearman’s correlation coefficient for tests with *p* value <0.05.

Our results showed that non-responders had a higher frequency of cells expressing IL-10 and TOX and fewer TCF-1 expressing cells, regardless of stimulus type (Figure S4). Specifically, higher levels of IL-10 expression were seen in non-responders among total CD4^+^ T lymphocytes, regulatory T lymphocytes, and EM CD4^+^ T lymphocytes (Figures 4A–4C). IL-10 production was widespread, as we observed IL-10-producing cells across all evaluated populations. The distribution of IL-10+ cells mirrored that of the lymphoid subpopulations. (Figure 4D). Conversely, responders had higher frequencies of TCF-1+ T cells in both CD4^+^ and CD8^+^ subpopulations (Figure 4E). Correlation matrices (Figure 4F) indicated a negative correlation between TCF-1+ T cells and T cells expressing the inhibitory molecules CTLA-4 or IL-10 in responders, suggesting an active immunoregulatory balance.

**Figure 5:**
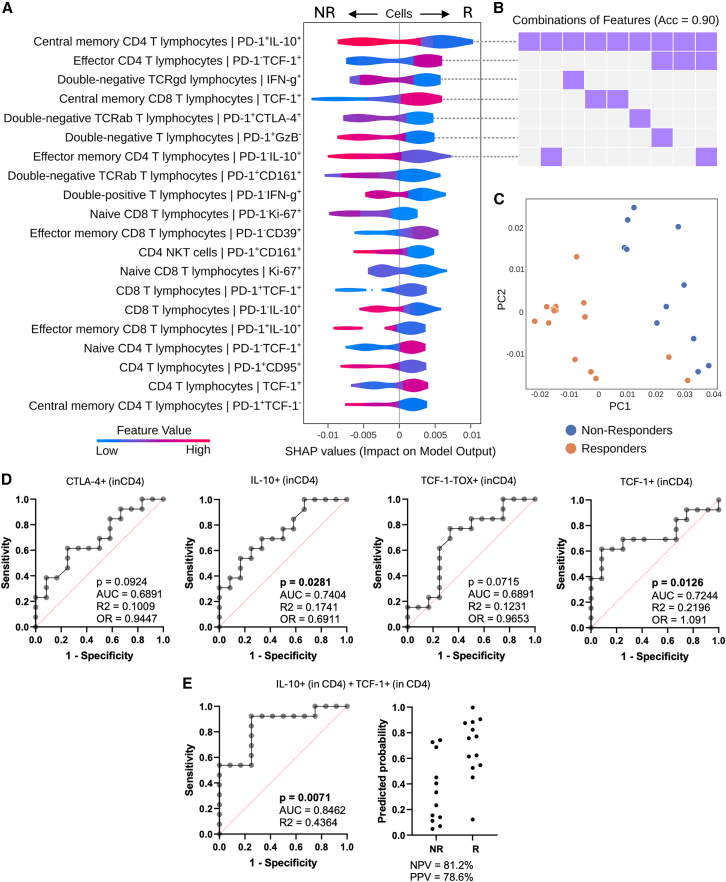
Relative importance of cell subpopulations for predicting responders (A) Violin plot shows the impact of each feature on model output (SHAP values). Positive SHAP values indicate the impact to predict responders, and negative SHAP values indicate the impact to predict non-responders. The features are sorted by Random Forest Importance from the top (most important) to the bottom (least important). (B) The feature combination analysis of the most important features. Each column represents a different feature set used to train a model. For all tests, the feature sets were 90% accurate in predicting responders. (C) Principal Component Analysis (PCA) using SHAP values of the cell types for responders and non-responders. (D and E) The accuracy of immune cell populations to predict response was assessed using ROC curves. (D) The accuracy of CTLA-4+, IL-10+, TCF-1+ or TOX+ CD4 T lymphocytes to predict response of NSCLC patients at the 9^th^ week of treatment (n=11 NR, n=13 R). AUC, p-value, Cox-Snell's R squared and odds ratio are shown after simple logistic regression analysis (1 parameter). (E) Combined analysis of the frequencies of IL-10+ and TCF-1+ CD4 T lymphocytes (n=11 NR, n=13 R). AUC, p-value, Nagelkerke's R squared and negative (NPV) or positive (PPV) predictive values are shown after multiple logistic regression analysis (2 parameters).

These findings align with the machine-learning unsupervised analysis. While the model using soluble factors alone yielded a 60% accuracy in distinguishing samples between responders and non-responders (Figures S1C and S1D), cytokines exhibited greater importance when considered in conjunction with cell types. The two most relevant cell types in distinguishing responders and non-responders were PD-1+IL-10+ CM CD4^+^ T lymphocytes and PD-1-TCF-1+ Eff CD4^+^ T lymphocytes (Figure 5A). In the RFC model, where responders served as the positive class, lower levels of PD-1+IL-10+ CM CD4^+^ T lymphocytes and higher values of PD-1-TCF-1+ Eff CD4^+^ T lymphocytes are related to the most critical features to identify responders, followed by TCRγδ lymphocytes expressing IFN-γ. Additionally, higher expression of TCF-1 was observed in CM CD8^+^ T cells, contributing positively to the identification of responders (Figure 5B). Notably, by evaluating different feature combinations, all sets of combinations included PD-1+IL-10+ CM CD4^+^ T cells with 90% accuracy (Figure 5B), indicating its strong predictive capability independent of other cell types. Further analysis of principal components using SHAP values from features in the violin plot, we can observe the ability of the model (Figure 5C) to differentiate between responder and non-responder samples.

We also observed an increased expression of immunosuppressive phenotype myeloid cells among non-responders as evidenced by the elevated frequency of IL-10 and PD-L2 in monocytes, macrophages, and dendritic cells (Figure S5). These results highlight the significance of IL-10 and TCF-1-expressing cell subpopulations as potential biomarkers of therapeutic response to immunotherapy in patients with NSCLC, as well as suggest immune mechanisms involved in differential responses.

In addition to the broad systemic immune cell populations and plasma metabolites identified earlier, specific subsets of CD4^+^ T lymphocytes, characterized by CTLA-4, IL-10, TCF-1, or TOX expression, were also predictive of response to immunotherapy (Figure 5D). A combination of CD4+IL-10+ and CD4+TCF-1+ T lymphocytes, using multivariate logistic regression analysis (Figure 5E), was the most effective predictor, achieving an AUC of 0.8462, R2 of 0.4364, and positive and negative predictive values of 78.6% and 81.2%, respectively. These results suggest that baseline frequencies of specific immune cell populations could be valuable predictive markers for treatment response in patients with NSCLC receiving immunotherapy.

**Figure 6:**
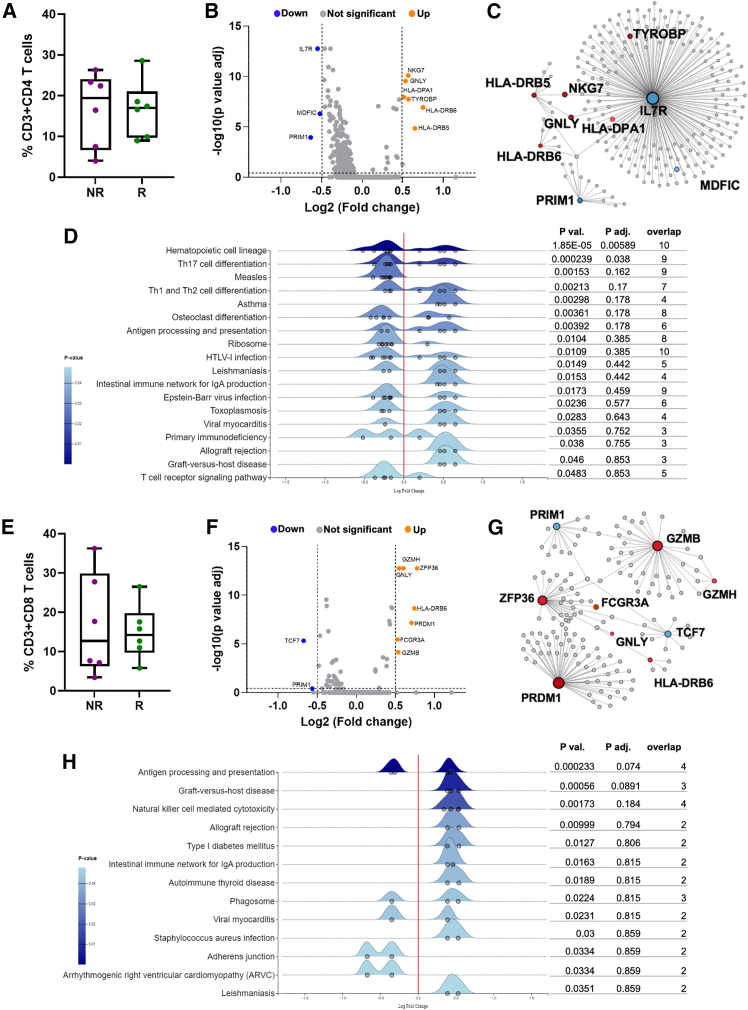
CD4^+^ and CD8^+^ T cells from responder and non-responder patients display distinct profiles as determined by scRNAseq analysis Peripheral blood samples from patients with NSCLC were collected before treatment initiation and processed for scRNAseq analysis as described in Methods. Non-responders (NR, purple symbols) or responders (R, green symbols) (*n* = 6 NR, *n* = 6 R) as defined at 9 weeks evaluation. (A–D) CD3^+^CD4^+^ (3,219 cells) and (E–H) CD3^+^CD8^+^ (2,196 cells) were analyzed separately. (A and E) Frequency of T cells from patients with NSCLC classified as non-responders or responders. Median and interquartile range are shown. (B and F) Volcano plot of responders compared to non-responders demonstrating differentially expressed genes (DEGs). Upregulated DEGs in responders (log2 fold-change ≥0.5 and *p* < 0.05) are shown in orange, and downregulated DEGs (log2 fold-change ≤ −0.5 and *p* < 0.05) are shown in blue. Genes without significant differential expression (log2 fold-change between 0.05 and −0.05 and *p* ≥ 0.05) are shown in gray dots. (C and G) Interaction network analysis of down (blue nodes) and upregulated (red nodes) genes in T cells from responders compared to non-responder (log(FC) value and *p* < 0.05). Node size reflects the number of protein interactions, shown as gray dots. Edges represent protein-gene interactions. (D and H) Top enriched signaling pathways in T cells from responders, based on DEGs. Pathways were identified via Kyoto Encyclopedia of Genes and Genomes (KEGG) analysis using the NetworkAnalysis tool, *p* < 0.005. Gray circles indicate DEGs, with overlaps showing the number of genes related to each pathway. Darker blue curves indicate lower *p* values.

With the aim of further delving into the cellular profiles and potential mechanisms associated with treatment outcome with anti-PD-1 and chemotherapy in patients with NSCLC, we performed single-cell RNA sequencing of the whole transcriptome of circulating CD4^+^ (3,219 cells) and CD8^+^ (2,196 cells) T cells from responders (*n* = 6) and non-responders (*n* = 6). No significant difference in the frequency of CD4 T cells and CD8 T cells between responder and non-responder patients with NSCLC was observed (Figures 6A and 6E). However, we found 9 differentially expressed genes (DEGs) in CD3^+^CD4^+^ T cells from responders, among which NKG7, GNLY, HLA-DPA1, TYROBP, HLA-DRB6, and HLA-DRB5 are upregulated, and IL7R, MDFIC, and PRIM1 are downregulated (Figure 6B). DEGs in CD4^+^ T cells from responders indicate that these cells are highly activated (expression of HLA class II molecules) and exhibit a cytotoxic profile (NKG7, GNLY), as well as the inhibition of markers associated with lung adenocarcinoma progression (IL7R) and mitotic division (PRIM1) in NSCLC. Network analysis demonstrates that these genes form a complex network of connections and are involved in enriching signaling pathways related to asthma, antigen processing and presentation, intestinal immune network for IgA production, viral myocarditis, allograft rejection, and graft-versus-host disease (Figures 6C and 6D).

The analysis of transcriptional profiles of CD8^+^ T cells from responders also identified 9 DEGs, among which GZMH, GZMB, ZFP36, GNLY, HLA-DRB6, PRDM1, and FCGR3A were upregulated, and TCF7 and PRIM1 were downregulated (Figure 6F). The upregulated genes in CD8^+^ T cells from responders are also associated with cytotoxic processes. PRDM1, when downregulated, is associated with cellular invasion and metastasis in lung cancer and inhibits TCF7, which indeed we found to be downregulated in this study. Interestingly, despite a higher frequency of cells expressing TCF-1 (protein expressed by the TCF7 gene), the expression level of this gene is reduced in responders, suggesting a potential negative feedback mechanism. Network analysis and signaling pathways show that DEGs in CD8^+^ T cells from responders are involved in enriching pathways related to graft-versus-host disease, NK cell-mediated cytotoxicity, allograft rejection, type 1 diabetes mellitus, intestinal immune network for IgA production, autoimmune thyroid disease, *Staphylococcus aureus* infection, and Leishmaniasis (Figures 6G and 6H).

To assess whether baseline clinical response was associated with antigen-driven clonal dynamics in the peripheral T cell compartment, we analyzed the TCR repertoire of circulating T cells prior to treatment initiation. TCR repertoire diversity, measured by the Shannon index, and the global distribution of clonotype sizes were comparable between responders and non-responders (Figures S6A and S6B). Clonotype expansion patterns across transcriptionally defined T cell subsets similarly showed no response-associated shifts (Figure S6C). UMAP projections of TCR-expressing CD4^+^ and CD8^+^ T cells revealed extensive transcriptional overlap between response groups, with no enrichment of expanded clonotypes in response-associated regions (Figures S6D and S6E). Functional transcriptional analyses further showed that CD4^+^ T cell activation, inactivation, and exhaustion gene program scores were similar between responders and non-responders and independent of clonotype size, whereas in CD8^+^ T cells, expanded clonotypes in responders exhibited higher exhaustion-associated gene program scores, a pattern less evident in non-responders (Figures S6F and S6G). Mean expression of individual genes within these programs did not reveal consistent response-associated differences. Collectively, these findings indicate that baseline peripheral TCR repertoire diversity and clonal expansion are not associated with clinical response, with response-associated differences instead reflected in functional transcriptional programs, particularly within expanded CD8^+^ T cell clonotypes.

### Peripheral blood T cells reflect the tumor microenvironment

While the tumor microenvironment (TME) is pivotal in dictating the antitumor response and therapy efficacy, the systemic immune response, as reflected in circulating blood, is also crucial. The analysis of peripheral blood immune subpopulations offers a unique view of immune cells activated in draining lymph nodes, now migrating toward the tumor site. This perspective is critical in understanding their activation states and functional potential, with direct relevance to clinical outcomes, as demonstrated in our current study.

**Figure 7:**
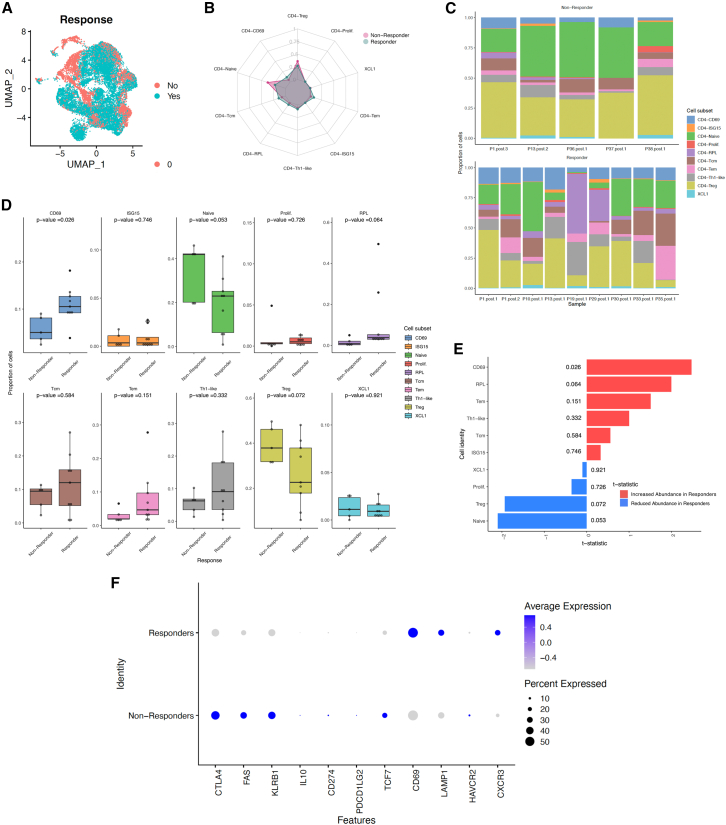
Single-cell RNAseq analysis of CD4 T lymphocyte subpopulation differences in response to immunotherapy for patients with NSCLC (A) UMAP plot reveals the distribution of CD4 T lymphocytes from responders (in teal) and non-responders (in red) (*n* = 5 NR, *n* = 9 R). (B) Radar plot provides an overview of the proportions of distinct CD4 T lymphocyte subpopulations in responders (in teal) and non-responders (in red) from all samples. (C) The stacked barplot shows the heterogeneity in the proportions of CD4 T lymphocyte subpopulations across all samples in both groups. (D and E) The boxplots (median and interquartile range are shown) and the horizontal barplot present the results of the differential abundance test. Boxplots depict the frequencies of each cell population, along with their respective *p* values comparing responders and non-responders. The horizontal barplot shows the log2 fold change in abundance, with red indicating increased abundance in responders and blue indicating reduced abundance in responders. (F) The dotplot illustrates the expression of markers of CD4 T lymphocyte subpopulations. Dot size corresponds to the percentage of cells expressing each gene marker in each group, and colors represent the average expression level. The CD4 T lymphocyte subpopulations are identified as CD4^−^CD69: CD69-expressing cells, CD4-ISG15: IFN stimulated genes-expressing cells, CD4-Naïve, CD4-Prolif: Proliferative cells, CD4-RPL: TCF7-expressing cells, CD4-Tcm: Central memory cells, CD4-Tem: Effector memory cells, CD4-Th1-like: CXCL13 and IFNG-expressing cells, CD4-Treg: Regulatory cells, XCL1: Chemokine-producer cells.

To corroborate our findings regarding peripheral blood T cell profiles in responding and non-responding patients with NSCLC, we utilized data from an independent cohort undergoing similar treatment regimens to our study (PD-1 blockade with chemotherapy, GSE179994). This dataset included scRNASeq from 9 responsive and 5 unresponsive tumor samples collected after the treatment.^17^ Our unsupervised analysis distinguished clear differences between the groups, revealing a higher proportion of regulatory and naive CD4^+^ T cells in non-responders. Conversely, responders showed an increased proportion of CD4^+^ T cells expressing CD69 (Figures 7A–7C and Table S3), aligning with our peripheral blood results.

Responder patients exhibited significantly greater levels of CD4^+^ T cells expressing CD69 (*p* value = 0.026), and an increase in the CD4RPL population, identified by a gene signature including TCF7, the gene for TCF1 studied using flow cytometry (*p* value = 0.064) (Figures 7D and 7E, and Table S3). In addition, responders had higher expression levels of CD69, LAMP1, and CXCR3, whereas non-responders had increased expression of CTLA4, FAS, and KLRB1 genes (Figure 7F). These results mirror what we found using flow cytometry with peripheral blood T cell subpopulations. For CD8^+^ T lymphocytes, a profile consistent with activation was observed in responders, despite a similar ISG-high CD8 state between responders and non-responders (Figure S7 and Table S3).

### CTLA-4 blockade partially restores T cell activation

Building on our finding that CTLA-4 is a key inhibitory molecule on CD4^+^ and CD8^+^ T lymphocytes in NSCLC non-responders treated with pembrolizumab (anti-PD-1) and chemotherapy, we initiated *in vitro* studies to evaluate if CTLA-4 blockade could modify their immune profiles. Non-responder patients’ PBMCs were stimulated with anti-CD3/CD28 in the presence of pembrolizumab (anti-PD-1), with or without ipilimumab (anti-CTLA-4).

**Figure 8:**
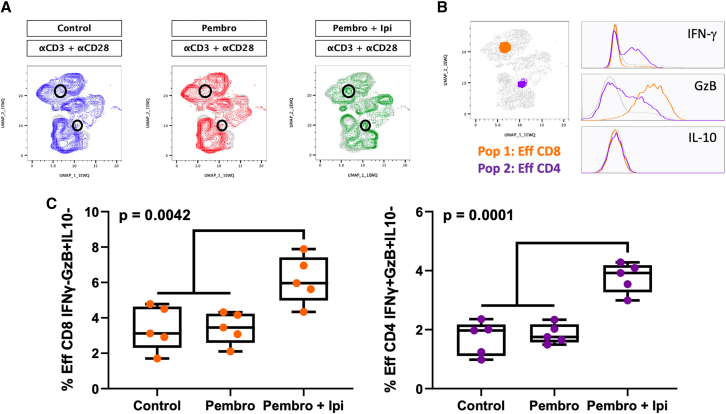
Ipilimumab partially restored T cell activation in non-responder patients with NSCLC treated with pembrolizumab plus chemotherapy PBMC from five non-responder patients were stimulated with pembrolizumab ± ipilimumab, in the presence of anti-CD3 and anti-CD28. (A) UMAP graphs for T lymphocyte populations. (B) Two lymphocyte populations differentially present in the two groups of patients were selected and analyzed for the expression of IFN-γ, granzyme B, and IL-10. (C) Frequency of T lymphocyte populations in each stimulus group. Median and interquartile range are shown. Statistical analysis using the Kruskal-Wallis test.

Our study demonstrated that dual blockade of CTLA-4 and PD-1 significantly augmented the production of IFN-γ and granzyme B in CD4^+^ effector T lymphocytes, and enhanced granzyme B in CD8^+^ effector T lymphocytes, while maintaining low IL-10 expression across both subsets (Figure 8). The importance of this finding is underscored by our unsupervised analyses (Figures 3A–3G), showing the importance of CTLA-4+ T lymphocyte subpopulations associated with non-responder patients. These results imply that CTLA-4 inhibition, in conjunction with PD-1 blockade, might effectively reinvigorate T cell activity and amplify anti-tumor responses in a subset of patients with NSCLC who initially did not respond to pembrolizumab and chemotherapy.

## Discussion

Immune checkpoint inhibitors, such as pembrolizumab, have markedly transformed cancer therapy, offering promising outcomes even in patients who are refractory to conventional treatments. In our study, focusing on patients with stage IV NSCLC treated with first-line pembrolizumab and chemotherapy, we observed an objective response rate of 42.5%. This finding aligns with prior reports,^4^^,^^5^^,^^6^^,^^7^ but also underscores the reality that a significant subset of patients (50–60%) does not benefit from this therapeutic approach. This discrepancy highlights the pressing need for reliable predictive biomarkers to forecast the efficacy of immunotherapy and to better understand the immune mechanisms behind differential clinical outcomes.

While PD-L1 expression is a validated biomarker for predicting immunotherapy response, its limited sensitivity and specificity pose challenges.^18^^,^^19^ Consequently, our study shifts focus to the systemic cellular immune response, particularly analyzing peripheral blood immune subpopulations. This analysis offers a glimpse into the activation state and functional potential of immune cells activated in draining lymph nodes, now migrating toward the tumor, providing crucial insights into mechanisms of clinical outcomes.

Our results indicate that elevated levels of total CD4^+^ T lymphocytes correlate with a lack of response at both 9 weeks (early response) and 6 months (durable response). This association suggests a potential suppressive role of CD4^+^ T cells in the anti-tumor immune response, resonating with earlier studies that reported an inverse relationship between the CD8+/CD4+ ratio in tumors and the response to anti-PD-1 therapy.^20^ Our data align with the notion that CD4^+^ T cells in non-responders may exhibit a more suppressive phenotype.^21^^,^^22^

In this context, the observation that responder patients were modestly older than non-responders warrants careful interpretation. Although immune aging is often linked to reduced adaptive immune function, clinical evidence in NSCLC suggests that age alone does not preclude benefit from immune checkpoint blockade, with outcomes strongly influenced by performance status and treatment selection.^23^ In our cohort, the age difference between responders and non-responders was limited in magnitude, supporting the interpretation that age may reflect broader host and immune context rather than acting as an isolated determinant of response.

Furthermore, we identified a relationship between a lower frequency of total CD4^+^ T lymphocytes and higher frequencies of activated CD4^+^CD69^+^ T lymphocytes or non-classical monocytes with prolonged PFS. This observation underscores the potential of these immune subpopulations as biomarkers for predicting therapeutic response and for guiding patient management. However, it is important to recognize that non-classical monocytes did not indicate a durable response, as their role appears to be more pertinent to initiating immune responses rather than maintaining them. Future research should focus on integrating these biomarkers with other factors to enhance the precision of predicting both early and long-term treatment outcomes in patients with cancer.

It is important to note that our study also sheds light on the role of CTLA-4 in response failure, a critical inhibitory regulator of T cell activation. CTLA-4 competes with the co-stimulatory receptor CD28 for binding to CD80/86 on APCs and is a hallmark of Treg and exhausted T cell subsets.^3^ Our findings show that non-responder patients have higher levels of CTLA-4+ CD4^+^ and CD8^+^ peripheral blood T lymphocytes before therapy begins, emphasizing the significance of this checkpoint in T cell dysfunction and immune escape in lung cancer.

In our analysis, a notable finding was the higher frequency of CD161+ and CD95^+^ T lymphocytes among non-responder patients with NSCLC. The role of CD161 in tumor progression remains complex, with literature suggesting it functions as both an activating and inhibitory receptor in T cells.^24^^,^^25^^,^^26^ Notably, the blockade of CD161 has been shown to enhance T cell-mediated killing of glioma cells, both *in vitro* and *in vivo*, suggesting a potential role for targeting CD161 in cancer immunotherapy.^26^^,^^27^ CD95, also known as Fas, is primarily recognized for its role in apoptosis but has also been implicated in inhibiting T cell receptor signaling and activation.^28^^,^^29^ These findings suggest that the upregulation of CD161 and CD95 in T lymphocytes from non-responder patients could represent mechanisms contributing to T cell dysfunction and tumor immune evasion. This is particularly relevant considering the observed higher expression of CTLA-4 in these cells, indicative of a suppressive T cell profile. Further research is warranted to validate these observations and explore the therapeutic targeting of these pathways in cancer immunotherapy.

Conversely, we identified elevated subpopulations of CXCR3 and TIM-3 expressing T cells in patients with NSCLC responding to immunotherapy. CXCR3, a chemokine receptor involved in T cell recruitment to inflamed tissues, has been associated with enhanced T cell responses.^30^^,^^31^ TIM-3, commonly viewed as an exhaustion marker on T cells,^32^^,^^33^ has also been detected on early activated T cells in various human diseases.^34^^,^^35^ Therefore, the presence of these markers is consistent with effective T cell activation en route to the TME.

The heatmap cluster analysis elucidated a potential interplay between T cell subpopulations favoring either response or non-response to immunotherapy. The first patient cluster, showing a durable therapeutic response (6 months), exhibited a higher frequency of T cells expressing CXCR3, CCR6, and/or TIM-3. This pattern suggests a more favorable immune response with important molecules related to tissue homing and potentially recent activation. In contrast, the second cluster, comprising non-responders, displayed a higher prevalence of T cells expressing the inhibitory molecules, CTLA-4, PD-1, and CD161, but lower levels of the beneficial T cell subsets seen in the responder group. Strikingly, the third cluster represents the early responder group, which displays a mixture of both the potentially beneficial and detrimental T cell subpopulations seen in the responders and non-responders, respectively. This dichotomy underscores the importance of the balance between inhibitory and activating signals in T cells, which appears to be a critical determinant of immunotherapy response in patients with NSCLC.

Upon investigating plasma concentrations of metabolites, we identified that non-responder patients with NSCLC exhibited elevated plasma concentrations of N-acetylneuraminate, taurodeoxycholate, xanthine, hypoxanthine, and AMP. These metabolites are noteworthy for their associations with immunosuppressive effects, potentially hindering anti-tumor immune responses. N-acetylneuraminate, for example, has been linked to increased aggressiveness and invasiveness in triple-negative breast tumors, as well as reduced antigenicity.^36^ Xanthine and hypoxanthine, both involved in adenosine metabolism, are compounds related to immunosuppression in the TME.^37^^,^^38^^,^^39^ Taurodeoxycholate, a bile acid, not only protects tumors from cytotoxicity by certain other bile acids, as suggested by its lower prevalence in responder patients,^40^ but also may contribute to angiogenesis, oxidative stress damage, p53 degradation, and invasiveness in various tumor types.^41^

Our study suggests a systemic reflection of an immunosuppressive response in non-responder patients with NSCLC, characterized by the co-expression of CTLA-4, CD161, and CD95 in immune cells and the production of immunosuppressive metabolites. Elevated IL-10 expression was noted in several circulating immune cell populations of non-responders. IL-10 is known to inhibit pro-inflammatory cytokines critical for T cell and NK cell activation, such as IL-12, TNF-alpha, and IFN-gamma, and suppresses dendritic cell differentiation and activation, key in tumor immune responses.^42^^,^^43^^,^^44^ Additionally, IL-10 can directly inhibit cytotoxic T lymphocytes and NK cells while promoting Treg, further dampening the anti-tumor response.^45^^,^^46^^,^^47^ Given these IL-10 expressing cells were identified in blood post-stimulation (polyclonal and SOX-2), it is plausible they were previously activated in tumor-draining lymph nodes and in transit to the tumor site expressing the homing chemokine receptor CCR6. This was reinforced through our analysis of the scRNAseq tumor samples from non-responders.

Conversely, responder patients exhibited a higher frequency of TCF-1-expressing immune cell populations. TCF-1 plays a vital role in controlling tumor development by promoting memory T cell formation and inhibiting the development of exhausted and dysfunctional CD4 and CD8 T cells.^48^^,^^49^^,^^50^ We also found a specific subset of activated CD4^+^ T lymphocytes, marked by CD69 expression, more prevalent among responders, indicating their potential as a response predictor. Activated CD4^+^ T cells can either directly kill tumor cells or activate CD8^+^ T cells and NK cells through Th1 cytokines.^51^^,^^52^ To further validate our findings, we performed single-cell RNAseq studies using PBMC from the same patient cohort. These studies showed the presence of activated CD4^+^ and CD8^+^ T lymphocytes with gene expression profiles consistent with cytotoxic function in the responder patients. Thus, two independent, yet complementary assays confirmed the findings in our cohort, along with the results from an independent cohort studying the TME gene expression profiles using scRNAseq, which also reinforced our findings.

Despite clear transcriptional differences between responders and non-responders identified by single-cell RNA sequencing, baseline TCR repertoire analyses did not reveal an association between global clonality metrics and treatment response. TCR repertoire diversity and overall clonal expansion were comparable between groups, indicating that response-associated transcriptional programs arise in the absence of major differences in peripheral repertoire architecture prior to therapy. This finding is consistent with our flow cytometry data, which point to functional immune states, rather than clonal dominance, as key features distinguishing responders from non-responders. While CD4^+^ T cell transcriptional programs were largely independent of clonotype size, expanded CD8^+^ T cell clonotypes in responders preferentially exhibited exhaustion-associated transcriptional signatures, a pattern consistent with antigen-experienced CD8^+^ T cell states previously associated with sensitivity to PD-1 pathway blockade and less evident in non-responders. The lack of a global clonality signal may reflect the limited sample size for TCR-informed analyses, the possibility that circulating TCR repertoires do not fully capture tumor-reactive dynamics occurring within the TME, and/or that clonal remodeling relevant to response emerges after treatment initiation rather than at baseline. Longitudinal analyses integrating paired blood and tumor samples will be required to further define the temporal and spatial contribution of T cell clonality to response and resistance in NSCLC.

This study highlights the critical role of systemic immune responses, as evidenced in peripheral blood, in reflecting therapeutic response and TME dynamics. Peripheral blood analysis of responder patients revealed cell populations with EM and effector profiles, possessing chemokine receptors and activation markers indicative of anti-tumor responses. In contrast, cells associated with therapy failure typically showed CM profiles with inhibitory receptors such as CTLA-4, PD-1, and CD161, and produced IL-10. Corroborating this, our independent NSCLC cohort analysis using scRNASeq showed distinct immune landscapes between responders and non-responders, with a higher proportion of CD4 T cells expressing CD69 and CXCR3 in responders and increased CTLA4, FAS, and KLRB1 gene expression in tumors from non-responders. These findings underscore the potential of peripheral blood immune signatures as indicators of TME dynamics and treatment response, as previously demonstrated,^53^^,^^54^^,^^55^ particularly in patients with NSCLC undergoing combined PD-1 blockade with chemotherapy.

Our research also revealed an association between non-classical monocytes and improved response to therapy at 9 weeks post-treatment initiation, although this correlation did not extend to 6 months. While these monocytes are generally linked to a suppressive profile in infectious diseases,^56^^,^^57^ their role in cancer remains ambiguous, oscillating between pro-tumoral actions, such as promoting angiogenesis and T cell suppression, and anti-tumoral activities, such as inducing cytotoxicity and preventing metastasis.^58^ Notably, previous studies have demonstrated that this monocyte subset can attract NK cells, thereby inhibiting tumor growth and reducing metastasis in lung cancer models.^59^^,^^60^ Their association with response at week 9 but not at 6 months may reflect a role in early immune remodeling during treatment initiation, whereas later outcomes may increasingly depend on acquired resistance and evolving suppressive mechanisms.

We observed an increased proportion of non-responders by 6 months, which is consistent with acquired resistance mechanisms. This temporal pattern suggests that baseline immune competence may influence early response and set the stage for subsequent outcomes that are also influenced by tumor evolution and treatment-induced immune remodeling.

The immune profiles investigated in our study shed light on the intricate mechanisms driving therapeutic responses in patients with NSCLC undergoing anti-PD-1 therapy combined with chemotherapy. A key highlight is the role of CTLA-4. Our functional assays indicated that co-blocking CTLA-4 and PD-1, using ipilimumab and pembrolizumab, respectively, partially reinvigorated T cell activation in cells from non-responder patients. This finding advocates for the potential effectiveness of a combined immunotherapy approach, especially in patients exhibiting elevated CTLA-4 expression in CD4^+^ and CD8^+^ T cells before undergoing treatment. We used polyclonal anti-CD3/CD28 stimulation in our functional assays. Although this approach is widely used to assess functional potential in antigen-experienced T cells and to reveal inhibitory checkpoint constraints, it cannot directly demonstrate tumor-antigen-specific reactivation. Antigen-specific testing was not feasible in the present cohort because matched tumor material and patient-level antigen expression data were not available, and additional paired biospecimens from the same patients were insufficient to support further assays. Larger studies with paired blood-tumor sampling and tumor-informed antigen selection will be important to address this question.

Supporting this notion, the phase III POSEIDON trial demonstrated significant improvements in both overall and PFS for metastatic patients with NSCLC treated with a combination of tremelimumab (anti-CTLA-4) and durvalumab (anti-PD-1), alongside chemotherapy, compared to those receiving chemotherapy alone.^61^ The data from this clinical trial did not conclusively show superior efficacy of the triple-drug regimen over the dual therapy of anti-PD-1 plus chemotherapy, since, such as other clinical trials, they compared immunotherapy regimens with standard chemotherapy and demonstrated an irrefutable superiority to chemotherapy alone,^62^^,^^63^ but did not compare between ICI (isolated or combined) regimes. Moreover, these studies did not propose biomarkers capable of directing patients to the best therapeutic approach based on response to therapy. In parallel, some studies have demonstrated the importance of combining anti-CTLA-4 with anti-PD-1, and have proposed potential mechanisms of action,^64^^,^^65^ however, they have focused on melanoma or have used *in vitro* or *in vivo* approaches rather than prospective patient cohorts. Our study advances the understanding of potential mechanisms of therapeutic failure in metastatic NSCLC treated with anti-PD-1 and chemotherapy by presenting a homogeneous cohort of patients naive to previous therapy, and provides evidence that patient stratification based on specific biomarkers on T cell subpopulations (e.g., CTLA-4) could enhance the likelihood of favorable responses to tailored immunotherapies.

Importantly, the biomarkers identified in this study were detectable in blood prior to therapy initiation, suggesting their utility in differentiating between effective and ineffective anti-tumor responses following checkpoint inhibitor therapy. This early detection offers a strategic advantage in tailoring treatments and pinpointing patients who are most likely to clinically benefit, especially in resource-constrained settings with limited access to immune checkpoint inhibitors such as Brazil.

In conclusion, our study has uncovered potential predictive biomarkers for immunotherapy response in patients with NSCLC, discernible in circulation and hence easily accessible (Figure S8). Unlike other biomarkers requiring invasive biopsies, these systemic markers offer a less invasive approach and potentially provide a more comprehensive representation of the overall anti-tumor response. In addition, we demonstrate immunosuppressive T cell subset profiles (e.g., CTLA-4+) that segregate patients based on therapeutic failure and can be used to identify individuals for the potential benefit of alternative therapeutic strategies. These findings bear significant clinical implications, paving the way for personalized treatment strategies that could improve patient outcomes.

### Limitations of the study

This study was conducted in a single-center cohort and, although it employed a multi-omics approach, it remains observational and hypothesis-generating. The proposed biomarkers require validation in larger, independent cohorts, and the limited availability of paired tumor samples restricted deeper exploration of tumor-immune interactions over time.

## Resource availability

### Lead contact

Requests for further information and resources should be directed to and will be fulfilled by the lead contact, Kennth J. Gollob (kenneth.gollob@einstein.br).

### Materials availability

This study did not generate new unique reagents.

### Data and code availability

• Data: Single-cell RNA-seq data have been deposited at GEO at GSE306542 accession number and are publicly available as of the date of publication (key resources table). Metabolomics raw data, including RT (min) and m/, is available as supplemental information (Table S6. Metabolomics raw data) and has been deposited at MetaboLights at MTBLS13150 accession number (https://www.ebi.ac.uk/metabolights/MTBLS13150) (key resources table).
• Code: All code used for the machine learning analysis is publicly available at: https://github.com/csbl-br/immunotherapy-response-circulating-cells/tree/main/ML_analysis.
• Any additional information required to reanalyze the data reported in this paper is available from the lead contact upon request.

## Acknowledgments

The authors would like to acknowledge the participation of patient volunteers, who made this study possible. This work was funded by FAPESP/GlaxoSmithKline grant (#2021/00408-6) and CNPq (#404186/2019-0). KJG, HTIN and WOD are CNPq research fellows. FAPESP also supports fellowships (#2018/06409-1, #2019/27139-5, #2018/14933-2).

## Author contributions

Conceptualization, A.B.F., V.C.C.L., and K.J.G.; methodology, A.B.F., K.H.M.C., V.M.C., R.B., R.P.L., H.T.I.N., W.O.D., and K.J.G.; experimentation, A.B.F., G.F.B.E., S.M.I.F., A.T.F., C.M.C., N.A.L.G., A.D.L., K.L.P.M., and I.P.S.; patient recruitment, classification and clinical care: L.M.K., H.C.F., J.L.G., C.A.L.P., R.C. and V.C.C.L.; formal analysis, A.B.F., A.T.F., T.G.S.S., J.C.S.S., and J.P.A.; resources, J.T.; all authors have reviewed the manuscript; writing – original draft, A.B.F. and K.J.G.; writing – review and editing: A.B.F., K.H.M.C., V.M.C., R.B., J.T., H.T.I.N., W.O.D., V.C.C.L., and K.J.G.; supervision and funding acquisition, K.J.G.

## Declaration of interests

This study was supported by a public-private partnership grant from FAPESP and GlaxoSmithKline (GSK) [grant #2021/00408-6]. GSK had no role in study design, data collection and analysis, decision to publish, or preparation of the manuscript. Two of the authors on this paper are employees of BD Biosciences, and antibodies and other reagents from BD Biosciences were used in the studies reported in this paper. No other authors have any potential conflict of interest.

## Declaration of generative AI and AI-assisted technologies in the writing process

During the preparation of this work, the authors used OpenAI’s ChatGPT 4o to assist in summarizing and refining parts of the manuscript text for clarity and conciseness. After using this tool, the authors reviewed and edited the content as needed and take full responsibility for the content of the publication.

## STAR★Methods

### Key resources table

**Table:** 

REAGENT or RESOURCE	SOURCE	IDENTIFIER
**Antibodies**		

Anti-human CD366 (TIM-3), 7D3, BV421	BD Horizon	Cat#565562; AB_2744369
Anti-human CD183 (CXCR3), 1C6/CXCR3, BUV395	BD Horizon	Cat#565223; AB_2687488
Anti-human CD294 (CRTH2), BM16, FITC	BD Pharmingen	Cat#561659; AB_10896659
Anti-human CD8, SK1, BUV805	BD Horizon	Cat#612889; AB_2833078
Anti-human CD152 (CTLA-4), BNI3, PE	BD Pharmingen	Cat#555853; AB_396176
Anti-human CD196 (CCR6), 11A9, PE-Cy7	BD Pharmingen	Cat#560620; AB_1727440
Anti-human CD223 (LAG-3), T47-530	BD Custom	N/A
Anti-human CD5, UCHT2, BV786	BD OptiBuild	Cat#740963; AB_2740588
Anti-human CD56, B159, APC	BD Pharmingen	Cat#555518; AB_398601
Anti-human TCRγδ, 11F2, APC-R700	BD Horizon	Cat#657707; AB_2870425
Anti-human CD19, SJ25C1, BV510	BD Horizon	Cat#562947; AB_2737912
Anti-human CD4, SK3, BV750	BD Horizon	Cat#566355; AB_2744426
Anti-human CD38, HB7, APC-H7	BD	Cat#653314; AB_2870353
Anti-human TCRVα24Jα18, 6B11, BV711	BD OptiBuild	Cat#747720; AB_2872199
Anti-human CD127, HIL-7R-M21, BB700	BD Horizon	Cat#566398; AB_2744279
Anti-human CD95, DX2, PE-CF594	BD Horizon	Cat#562395; AB_11153666
Anti-human CD25, 2A3, BUV563	BD Horizon	Cat#612918; AB_2870203
Anti-human CD279 (PD-1), EH12.1, BUV737	BD Horizon	Cat#612791; AB_2870118
Anti-human CD39, TU66, BV605	BD OptiBuild	Cat#742522; AB_2740840
Anti-human CD197 (CCR7), 2-L1-A, BV650	BD Horizon	Cat#566756; AB_2869851
Anti-human CD161, DX12, PE-Cy5	BD Pharmingen	Cat#551138; AB_394068
Anti-human CD3, UCHT1, BUV661	BD Horizon	Cat#569198; AB_3684860
Anti-human CD45, HI30, BB790	BD Custom	N/A
Anti-human CD185 (CXCR5), RF8B2, BB660	BD Custom	N/A
Anti-human CD45RO, UCHL1, BV570	BD Custom	N/A
Anti-human IL-17A, N49-653, BV421	BD Horizon	Cat#562933; AB_2737902
Anti-human TNF-α, MAb11, BUV395	BD Horizon	Cat#563996; AB_2738533
Anti-human Foxp3, 259D/C7, Alexa Fluor 488	BD Pharmingen	Cat#560047; AB_1645349
Anti-human TOX, TXRX10, PE	ThermoFisher	Cat#12-6502-82; AB_10855034
Anti-human Ki-67, B56, PE-Cy7	BD Pharmingen	Cat#561283; AB_10716060
Anti-human TCF-1, S33-966, BUV615	BD Custom	N/A
Anti-human Granzyme B, GB11, BV510	BD Horizon	Cat#563388; AB_2738174
Anti-human IL-10, JES3-9D7, BV711	BD Horizon	Cat#564050; AB_2738564
Anti-human IFN-γ, B27, BV605	BD Horizon	Cat#562974; AB_2737926
Anti-human CD11c, B-ly6, BV421	BD Horizon	Cat#562561; AB_2737656
Anti-human CD169 (Siglec-1), 7–239, BB515	BD Horizon	Cat#565353; AB_2739204
Anti-human CD66b, G10F5, Alexa Fluor 647	BD Pharmingen	Cat#561645; AB_10894001
Anti-human CD14, M5E2, BUV805	BD Horizon	Cat#612902; AB_2870189
Anti-human CD16, 3G8, Alexa Fluor 700	BD Pharmingen	Cat#557920; AB_396941
Anti-human IL-10, JES3-9D7, PE	BD Pharmingen	Cat#554498; AB_395434
Anti-human CD206, 19.2, PE-Cy7	BD Custom	N/A
Anti-human CD86, 2331 (FUN-1), BUV615	BD Custom	N/A
Anti-human CD68, Y1/82A, BV786	BD Custom	N/A
Anti-human CD15, W6D3, BV510	BD Horizon	Cat#563141; AB_2738025
Anti-human CD19, SJ25C1, BV750	BD OptiBuild	Cat#747161; AB_2871897
Anti-human CD141, 1A4, BV711	BD Horizon	Cat#563155; AB_2738033
Anti-human CD1c, F10/21A3, BB700	BD OptiBuild	Cat#746095; AB_2743468
Anti-human CD117 (ckit), YB5.B8, PE-CF594	BD Custom	N/A
Anti-human CD2, RPA-2.10, BUV737	BD OptiBuild	Cat#741821; AB_2871156
Anti-human FcεR1, ERA-37, BV605	BD Custom	N/A
Anti-human CD163, GHI/61, BV650	BD Horizon	Cat#563888; AB_2738468
Anti-human CD123, 9F5, PE-Cy5	BD Pharmingen	Cat#551065; AB_394029
Anti-human HLA-DR, G46-6, BUV661	BD Horizon	Cat#612980; AB_2870252
Anti-human CD274 (PD-L1), MIH1, BB630	BD Custom	N/A
Anti-human CD273 (PD-L2), MIH18, BB660	BD Custom	N/A
Anti-human CD80, L307.4, BB755	BD Custom	N/A
Anti-human CD11b, ICRF44, BV570	BD Custom	N/A
Ultra-LEAF Purified Human IgG4 Isotype Control	BioLegend	Cat#403701
Ultra-LEAF Purified Human IgG1 Isotype Control	BioLegend	Cat#403501
Pembrolizumab, Keytruda	MSD	N/A
Ipilimumab, Yervoy	Bristol-Myers Squibb	N/A
Purified NA/LE anti-human CD3, HIT-3α	BD Biosciences	Cat#555336; AB_395742
Purified NA/LE anti-human CD28, CD28.2	BD Biosciences	Cat#555725; AB_396068

**Chemicals, peptides, and recombinant proteins**		

SOX-2 overlapping peptides	In-house	N/A

**Critical commercial assays**		

Bio-Plex Pro Human Cytokine 48-Plex Screening Panel	Bio-Rad	Cat#12007283
Cartridge Kit	BD Rhapsody	Cat#673733
Enhanced Cartridge Reagent Kit	BD Rhapsody	Cat#664887
cDNA Kit	BD Rhapsody	Cat#633773
WTA Amplification Kit	BD Rhapsody	Cat#633801
TCR/BCR Amplification Kit	BD Rhapsody	Cat#665345
Human Single-Cell Multiplexing Kit	BD Rhapsody	Cat#633781

**Deposited data**		

scRNASeq available data	GEO-NCBI	GSE179994
scRNASeq new data	GEO-NCBI	GSE306542
Metabolomics raw data	MetaboLights	MTBLS13150

**Software and algorithms**		

FlowJo	BD Biosciences	v.10.8
SevenBridges	Velsera	BD Rhapsody Sequence Analysis Pipeline v.0
SeqGeq	BD Biosciences	v.1.8.0
Prism	GraphPad	v.8.4
Seurat	SatijaLab	v.4.0.5; v.4.3.0.1
Code for Machine Learning Analysis	Github	https://github.com/csbl-br/immunotherapy-response-circulating-cells/tree/main/ML_analysis

### Experimental and study participant details

All patients were treated under routine clinical care at A. C. Camargo Cancer Center, São Paulo, Brazil. The diagnosis, treatment and monitoring of patients were carried out regardless of any inclusion or exclusion criteria. This study was approved by the local Ethics Committee at AC Camargo Cancer Center under number 2463/17, registered with the national Brazilian ethics committee authority, CONEP. Written informed consent was obtained from each patient before the initiation of any procedure, in accordance with the Brazilian CNS #466 Resolution. We included patients of both sexes aged 18 years or over, histologically confirmed diagnosed of stage IV NSCLC that were treated with the combination of pembrolizumab + chemotherapy. Patients with non-squamous NSCLC received 4 cycles of carboplatin AUCx6 + pemetrexed 500 mg/m2 + pembrolizumab 200 mg once every 3 weeks, followed by pembrolizumab 200 mg for up to 35 cycles. Patients with squamous NSCLC received 4 cycles of carboplatin AUCx6 + paclitaxel 200 mg/m2 + pembrolizumab 200 mg once every 3 weeks, followed by pembrolizumab 200 mg for up to 35 cycles. Individual clinical characteristics for the patient cohort is shown in Table S4. We excluded: patients diagnosed with lung cancer other than NSCLC, patients with a previous diagnosis of another invasive cancer within 5 years, patients using antibiotic therapy, corticosteroids, immunosuppressive drugs or NSAIDs, and patients who received any previous treatment for the metastatic disease, including surgery, radiotherapy, immunotherapy, targeted therapy or any experimental therapy. Patients with disease progression were included in the “non-responder” group and patients with complete or partial response or stable disease were included in the “responder” group. Responses were assessed 9 weeks or 6 months after starting treatment. Images (contrast-enhanced computed tomography and brain MRI) were evaluated for response according to RECIST1.1 criteria. Responses were reviewed by two experienced thoracic oncologists (VCCC and HCF).

### Method details

#### Blood samples

Peripheral blood was collected in EDTA tubes at the time of diagnosis, before starting the treatment. Whole blood aliquots were evaluated by flow cytometry. Blood samples were centrifuged within 2–4 h after collection and the plasma was frozen immediately at −80°C. Plasma samples were used for metabolomics analysis. Remained blood was diluted 1:1 with PBS, PBMC were purified using a Ficoll gradient and cryopreserved in FBS/DMSO.

#### Soluble factors multiplex assay

We measured 48 substances in the patients' plasma to evaluate the soluble immune profile, including inflammatory and anti-inflammatory mediators, and growth factors. We used the Bio-Plex Pro Human Cytokine Screening Panel kit on the Bio-Plex platform (Bio-Rad) according to manufacturer’s instructions. The following proteins were measured: basic FGF, CTACK (CCL27), eotaxin (CCL11), G-CSF, GM-CSF, GRO-α (CXCL1), HGF, IFN-α2, IFN-γ, IL-1α, IL-1β, IL-1ra, IL-2, IL-2Ra, IL-3, IL-4, IL-5, IL-6, IL-7, IL-8, IL-9, IL-10, IL-12p40, IL- 12p70, IL-13, IL-15, IL-16, IL-17A, IL-18, IP-10 (CXCL10), LIF, MCP-1 (CCL2), MCP-3 (CCL7), M-CSF, MIF, MIG (CXCL9), MIP-1α (CCL3), MIP-1β (CCL4), β-NGF, PDGF-BB, RANTES (CCL5), SCF, SCGF-β, SDF-1α (CXCL11), TNF-α, TNF-β, TRAIL, VEGF-A.

#### Flow cytometry

The systemic cellular immune profile was characterized by multiparametric flow cytometry using fresh whole blood or cryopreserved PBMC. All comparisons were made using the same sample type and we did not analyze fresh or frozen cells together. Whole blood samples were incubated with antibodies (Table S5) for 15 min at room temperature, followed by the lysis of red blood cells (BD FACS Lysing Solution, BD Biosciences). Samples were fixed in PBS/2% formaldehyde and stored at 4°C until the moment of acquisition. PBMC were thawed, confirmed for viability using trypan blue exclusion, and incubated in culture medium overnight at 37°C/5% CO_2_ under the following conditions: (1) unstimulated; (2) anti-CD3 NA/LE (HIT-3α clone, BD Biosciences) + anti-CD28 NA/LE (CD28.2 clone, BD Biosciences); and (3) SOX-2 overlapping peptides + anti-CD28. All stimuli were used at 1 μg/mL. After overnight incubation, cells were washed and resuspended in stain buffer for viability evaluation (Live/Dead Fixable Blue UV, ThermoFisher) and incubation with the appropriate collection of antibodies (Table S5). The cells were fixed and permeabilized to investigate intracellular markers using the BD Pharmingen Transcription Factor Buffer Set (BD Biosciences). All panels were developed with BD Biosciences, tested and confirmed before use in this study to generate compensation matrixes and other instrument settings which were maintained for every sample. At least 200,000 gated cells were acquired using the BD FACSymphony A5 flow cytometer. Analyses were performed using FlowJo software version 10.8. Analysis strategies are shown in Figures S9–S11.

#### Metabolomics analysis

The metabolic profile was evaluated in plasma. The analytes were separated by ultra-performance liquid chromatography (UPLC, ACQUITY UPLC I-Class, Waters Corporation) and analyzed by high resolution mass spectrometry (Q Exactive HF-X, hybrid quadrupole orbitrap mass spectrometer, Thermo Fisher Scietific). The analysis strategy was untargeted metabolomics, in which different classes of compounds were considered, such as amino acids, lipids and nucleotides. Fifty microliters of plasma were transferred to wells of a 96-well plate followed by 12 μL of internal standard mixture, composed of tryptophan-d_5_, muconic acid-d_4_, methionine-d_3_ and androstenedione-d_7_ at 2 μmol/L, phenylalanine-d_5_ at 0.4 μmol/L, oleic acid-13C_18_ at 6 μmol/L and succinic acid-d_4_ at 1 μmol/L in water. The plate was mixed for 5 min at 700 rpm in a Thermomixer (Eppendorf, Hamburg, Germany). To each well, 200 μL of a cold solution of methanol:isopropanol 1:1 (v/v) were added. The plate was mixed for 15 min at 1400 rpm and 4 °C. Then, the plate was centrifuged for 30 min (3700 rpm, 4 °C) and 175 μL of supernatant was collected. The supernatant was dried and reconstituted in 50% acetonitrile. Plasma extract was then filtered with AcroPrep GHP 0.2 μm (Pall Corporation, Port Washington, NY, USA) by centrifugation at 1000 rpm for 5 min. A volume of 30 μL of the filtered extract was transferred to another plate and 170 μL of water was added. The mixture was homogenized for 5 min at 700 rpm. A pool of non-responders, a pool of responders and healthy volunteers were extracted in an identical way and were analyzed at the beggining, middle and end of the worklist as quality control samples. Metabolomics analysis was performed on an Acquity UPLC I-Class (Waters Corporation, Milford, MA, USA) LC systems coupled to a Q-Exactive HF-X mass spectrometer (Thermo Fisher Scientific, Waltham, MA, USA) equipped with a heated electrospray ionization (HESI) source operating in positive and negative ion mode. A 5 μL aliquot of extracted plasma was injected onto an Acquity UPLC CSH C18 1.7 μm, 100 × 2.1 mm column (Waters Corporation) protected by a UHPLC C18 4 × 2 mm precolumn (Phenomenex, Torrance, CA, USA), both maintained at 40°C. Mobile phase A was composed of 0.1% formic acid in water and mobile phase B was composed of 0.1% formic acid in acetonitrile. Separation was performed by gradient elution: 1% B (0–2 min), 1–99% B (2–13 min), 99% B (13–16 min), 99-1% B (16–16.1 min), 1% B (16.1–17 min). Flow rate was 0.3 mL/min. MS source parameters was set as follows: capillary temperature = 320°C for negative mode and 275°C for positive mode, capillary voltage = 3.5 kV, nitrogen sheath and auxiliary gas 47.5 and 11.25 arbitrary units, respectively, S-Lens RF level = 50 and mass range from 70 to 800 *m/z*. Full MS experiment was acquired with a resolution of 30,000 for negative mode and 60,000 for positive mode, AGC target = 5e^6^ for negative mode and 3e6 for positive mode and maximum IT = 64 ms for negative mode and 128 ms for positive mode. DIA acquisition was based in 23 windows of 31 Da width *m/z* precursor windows at 15,000 resolution, AGC target 5e,^6^ maximum IT 64 ms, and normalized collision energy (NCE) 60, covering 50–800 m/z in MS1 and 70.5–761.5 in MS2. Data pretreatment was performed on MS-DIAL (version 4.12).^66^ Median normalization was applied before statistical analysis. Only molecular features present at least 70% in one group and with the coefficient of variation lower than 30% in one quality control group was considered for statistical analysis. Metabolite identification was accomplished by matching retention time and deconvoluted MS/MS data with an *in house* library established with authentic analytical standards from MSMLS library (Sigma-Aldrich, San Luis, MO, EUA) or by matching deconvoluted MS/MS data with public libraries such as Mass Bank of North America (https://mona.fiehnlab.ucdavis.edu/) and Metlin.^67^

#### Single-cell RNA sequencing

In house scRNASeq experiments were performed using the BD Rhapsody Express Single-cell Analysis System platform, according to the manufacturer’s instructions. PBMC samples were previously sorted using BD FACS Aria Fusion for live leukocytes and labeled with nucleotide-conjugated antibody sample tags (Human Single-Cell Multiplexing Kit, BD Rhapsody). Briefly, the cell suspension was applied to the cartridges at a suitable dilution and speed so that a single cell was distributed per well, where beads were distributed in sequence. Each bead had a specific barcode, in addition to a poly-T tail for capturing mRNA transcripts released after cell lysis. Then, reverse transcription, amplification and purification steps were performed to construct a cDNA library (Cartridge, Enhanced Cartridge Reagent, cDNA, WTA Amplification and TCR/BCR Amplification Kits, BD Rhapsody), which was submitted to deep sequencing in the NovaSeq 6000 equipment (Illumina). Data processing was initially conducted through the Seven Bridges Genomics platform, which incorporated the BD Rhapsody Sequence Analysis Pipeline. Processed data in MTX format were imported into SeqGeq version 1.8.0. Sample-normalized and quality-controlled cells were submitted to the Seurat plugin version 4.0.5, with identification of highly dispersed genes. Differentially expressed genes were identified using the FindMarker function, applying a log2 fold change threshold (≥0.5 or ≤ −0.5) and a *p* value <0.05. Graphs were constructing using SRplot web tool.^68^

T cell receptor (TCR) repertoire analysis was performed using Scirpy (v0.22.4) applied to AIRR-formatted V(D)J sequencing files. A total of 7,034 cells with detectable V(D)J sequences were identified. Transcriptomic and V(D)J data were integrated into a single annotated object, and only cells with corresponding gene expression profiles were retained for downstream analyses. Cells expressing orphan chains, ambiguous receptor assignments (simultaneous TCR and BCR), or multiple TCR chains were excluded, resulting in 2,229 good-quality cells with functional V(D)J chains. 1,096 TCR-expressing T cells were identified based on the combination of transcriptomic cell type annotation and TCR expression.

Clonotypes were defined based on amino acid (CDR3) sequence similarity across all receptor arms, considering only primary chains in the presence of dual immune receptors. TCR repertoire diversity was quantified using the Shannon diversity index calculated per patient. Differences in clonotype diversity between clinical response groups were assessed using a two-sided Mann–Whitney U test, with statistical significance defined as *p* ≤ 0.05. Clonotype expansion metrics were normalized by the total number of TCR-expressing cells per sample.

Cell-level quality control was conducted following the median absolute deviation (MAD)–based strategy (MAD = 5), and outlier cells were removed accordingly. Cells with a more that 20% of mitochondrial genes were also excluded. Genes expressed in fewer than 10 cells were filtered out. After quality control, a total of 22,490 high-quality cells and 21,298 genes were retained for downstream analyses.

Filtered counts were normalized to total counts per cell, followed by log1p-transformation. Highly variable genes (HVGs) were identified accounting for patient-level effects. Batch correction was performed using Harmony (v 0.0.10) integration with batch defined by the experimental batch variable. A k-nearest neighbor graph was constructed using the Harmony-corrected PCA representation, with the number of principal components selected based on explained variance. Nonlinear dimensionality reduction was performed using Uniform Manifold Approximation and Projection (UMAP; v0.5.7).

Cell clustering was carried out using the Leiden algorithm (v0.10.2) with a resolution parameter of 1.0, resulting in 13 clusters. Annotation of cell populations was performed using Celltypist (v1.7.1) with the pretrained Immune_All_Low reference model, applying majority voting to improve annotation robustness.

#### *In vitro* PBMC stimulation

For PBMC culture, cells from five non-responder patients were thawed, confirmed for viability using trypan blue exclusion, and incubated in culture medium at 37°C/5% CO_2_ with 10 μg/mL pembrolizumab (anti-PD-1) ±10 μg/mL ipilimumab (anti-CTLA-4) or their respective isotype controls. After 30 min, cells were stimulated with anti-CD3 NA/LE and anti-CD28 NA/LE, both at 1 μg/mL. After overnight incubation, cells were washed and resuspended in the staining solution with the appropriate mixture of antibodies, as described above.

#### Machine learning analysis

To evaluate the feature contribution in predicting response at nine weeks, we conducted a machine-learning classification analysis with Responders vs. Non-responders using responders as the positive class. This analysis used a Random Forest Classifier (RFC) from Scikit-learn python library v1.2.0.^69^ We independently analyzed clinical, metabolite, cytokine and cell type datasets. Preprocessing of each dataset involved a pipeline to handle categorical and numerical features. Categorical features were transformed into dummy variables while numerical features were normalized using the StandardScaler function to achieve unit variance. The datasets were split into 70% training samples (*N* = 23) and 30% testing samples (*n* = 10) while ensuring stratification. The RFC hyperparameters were tuned using the GridSearchCV function with 5-fold cross-validation. To quantify the positive and negative contributions of each feature on the prediction, we employed the Shapley Additive Explanations (SHAP) technique from the shap python library v.0.41.0.^70^ For cell types, we performed a feature combination analysis constructing models for every set of combinations for the top 10 most important features. Only the topmost accurate combinations were considered. Additionally, we employed Principal Component Analysis using SHAP values to investigate how patients are clustered based on the features analyzed.

#### scRNASeq public data analysis

The raw counts matrix for scRNA-seq was downloaded from the GEO database (GSE179994), along with the corresponding metadata. Data from 9 responsive and 5 unresponsive tumor samples collected post-treatment were used.^17^ A Seurat object was created, and initial filtering was applied to retain genes expressed in at least 3 cells and cells with a minimum of 200 expressed genes. Quality control was performed by excluding cells with fewer than 600 expressed genes, more than 25,000 expressed genes, or fewer than 600 UMIs. Following preprocessing, further analysis was conducted using the Seurat (version 4.3.0.1) standard workflow (detailed in https://satijalab.org/seurat/archive/v4.3/pbmc3k_tutorial). For the characterization of CD4 and CD8 lymphocyte subpopulations, annotations from the original study were used. Gene markers defining these subpopulations are detailed in Extended Data Figures 2 and 3 of the original publication (https://www.nature.com/articles/s43018-021-00292-8#Sec9). After this initial characterization, CD4 T and CD8 T lymphocytes were separately subset from post-immunotherapy samples. To identify low-quality cells, the isOutlier() function from the scuttle package (version 1.10.2) was used, with thresholds set at 2 median absolute deviations (MADs) from the median. Cells outside this threshold were flagged as low-quality. Quality control was performed independently for CD4 T and CD8 T post-treatment populations (https://genomebiology.biomedcentral.com/articles/10.1186/s13059-020-02136-7). Once preprocessing and clustering were complete, differential abundance analysis was performed on CD4 and CD8 T lymphocyte subpopulations, comparing responders to non-responders to immunotherapy. The scanpro package (version 0.3.2) was used for this analysis, considering *p*-values <0.05 as statistically significant(https://www.nature.com/articles/s41598-024-66381-7, https://github.com/loosolab/scanpro). For visualization, the fsmb package (version 0.7.5) and ggplot2 package (version 3.4.3) were used. All analyses were performed using R (version 4.3.0).

#### Statistical analysis

Gaussian distribution was evaluated by Shapiro-Wilk test. For comparisons between groups, Mann-Whitney-Wilcoxon test or Student’s *t* test were used when appropriate. Heatmaps were constructed using ClustVis web tool.^71^ To assess the accuracy of markers as predictors of response, receiver operating characteristic (ROC) curves were generated, followed by simple or multiple logistic regression. Finally, progression-free survival (PFS) curves were calculated by the Kaplan-Meier method and compared with the Log Rank test (Mantel-Cox). All analyses were performed using GraphPad Prism software version 8.4. *p*-values <0.05 were considered statistically significant.

## Supplemental information

Document S1. Figures S1–S10, and Tables S1–S5

Table S6. Metabolomics raw data
