# Supplementary material for: Circulating cell populations as response predictors and targets to improve immunotherapy in metastatic lung cancer
Source: iScience. 2026 Jun 8;29(6):116124. doi: 10.1016/j.isci.2026.116124 (PMC13264123; doi:10.1016/j.isci.2026.116124)
Supplement: Document S1. Figures S1–S10, and Tables S1–S5 [file mmc1.pdf]

iScience, Volume *29*

Supplemental information

Circulating cell populations as response predictors and targets to improve immunotherapy in metastatic lung cancer

Amanda B. Figueiredo, Guilherme F.B. Evangelista, Stephanie M.I. Ferreira, Larissa M. Kuil, Gabriela Barbeta, Lukas C. Iohan, Andrea T. Faccio, Karina H.M. Cardozo, Valdemir M. Carvalho, Clara M. Cavalcanti, Robert Balderas, Rodrigo P. Lopes, James Turner, Thaiany G. Souza-Silva, Juan C.S. Silva, Jonathan P. Avila, Helder T.I. Nakaya, Nayane A.L. Galdino, Ananda D. Lopes, Kátia L.P. Morais, Iasmim P. Santos, Helano C. Freitas, Jefferson L. Gross, Clóvis A.L. Pinto, Rubens Chojniak, Walderez O. Dutra, Vladmir C. Cordeiro-de-Lima, and Kenneth J. Gollob

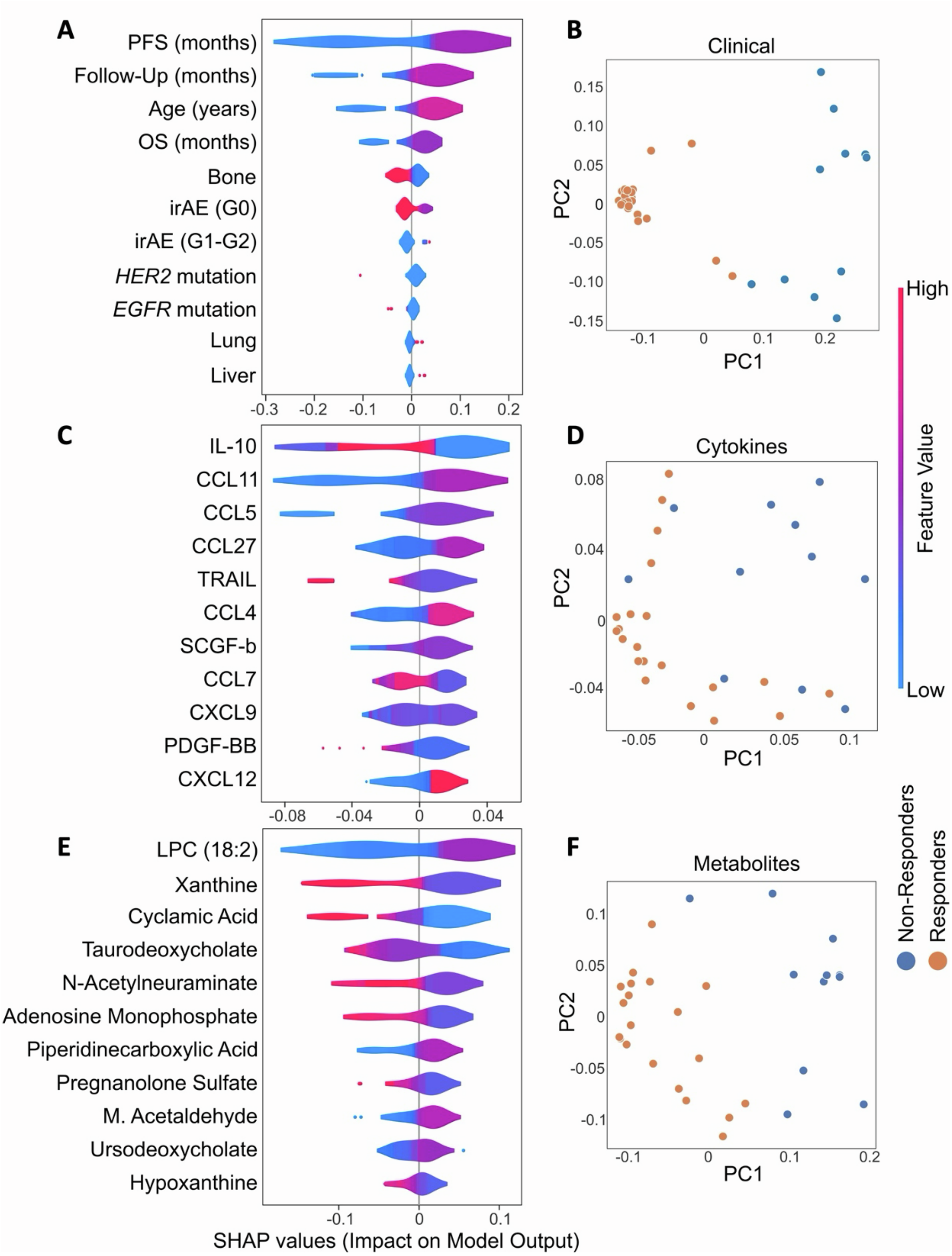

Figure S1. Feature importance for predicting responders, Related to Study participant details STAR Methods. A violin plot and principal components from SHAP values were analyzed for clinical (**A-B**), cytokines (**C-D**) and metabolites (**E-F**). The violin plots (**A-C-E**) show the impact of each feature on model output (SHAP values). Positive SHAP values indicate the impact to predict responders and 0ative SHAP values indicate the impact to predict non-responders. The features are sorted by Random Forest Importances from the top (most important) to the bottom (less important). The low (blue) and high (red) feature values represent the relatively lower and higher values of the feature itself (e.g. range of age from 26.2 to 80.8 years). The Principal Component Analysis (PCA) using SHAP values (**b-d-f**) was performed for all datasets considering responders and non-responders samples (n=11 NR, n=22 R).

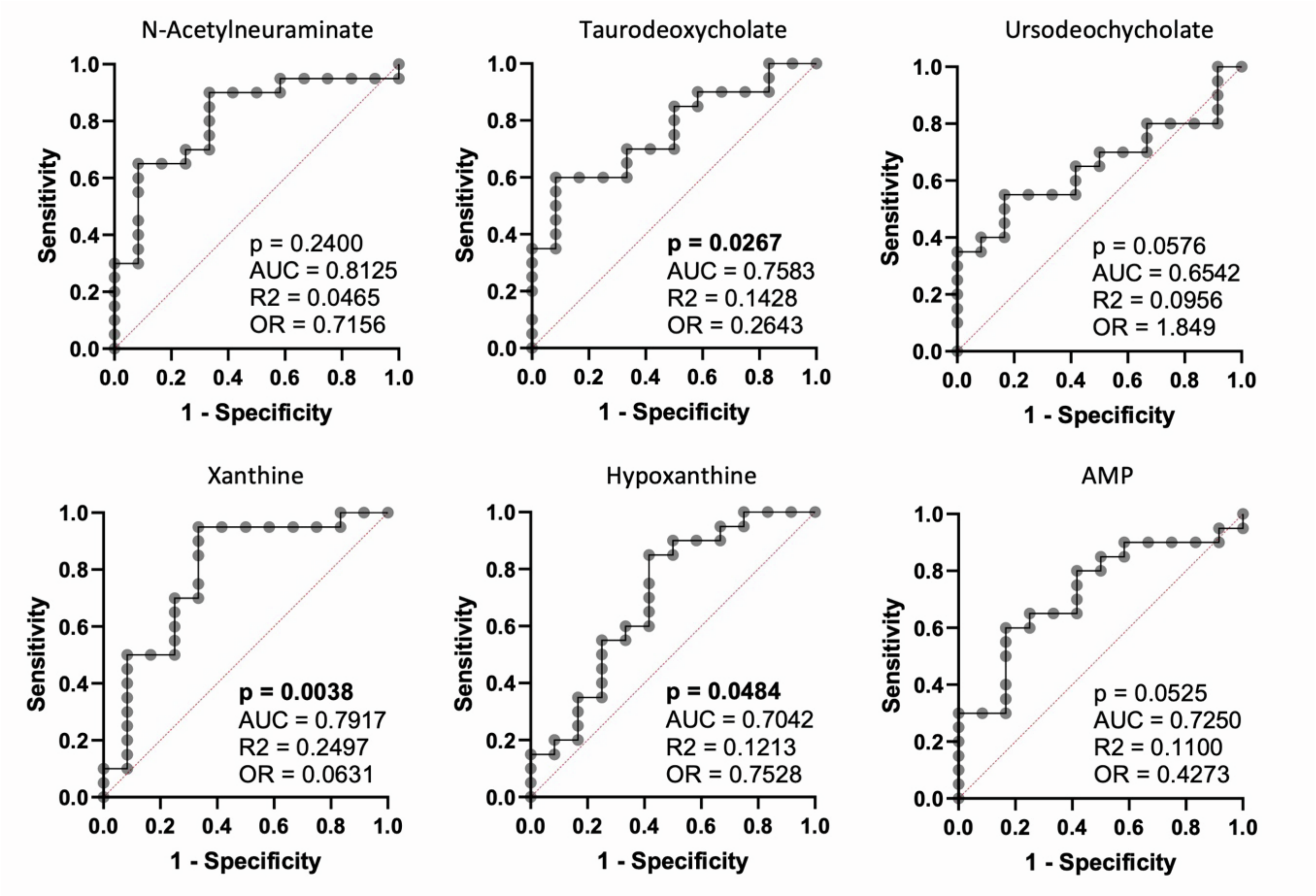

Figure S2. Metabolites predict response to immunotherapy for NSCLC patients, Related to Figure 1. Plasma samples were analyzed by ultra-performance liquid chromatography and high-resolution mass spectrometry, and the relative amount of metabolites was compared between non-responder and responder groups (n=11 NR, n=21 R). The accuracy of these metabolites to predict response was assessed using ROC curves. AUC, p-value, Cox-Snell's R squared and odds ratio are shown after simple logistic regression analysis.

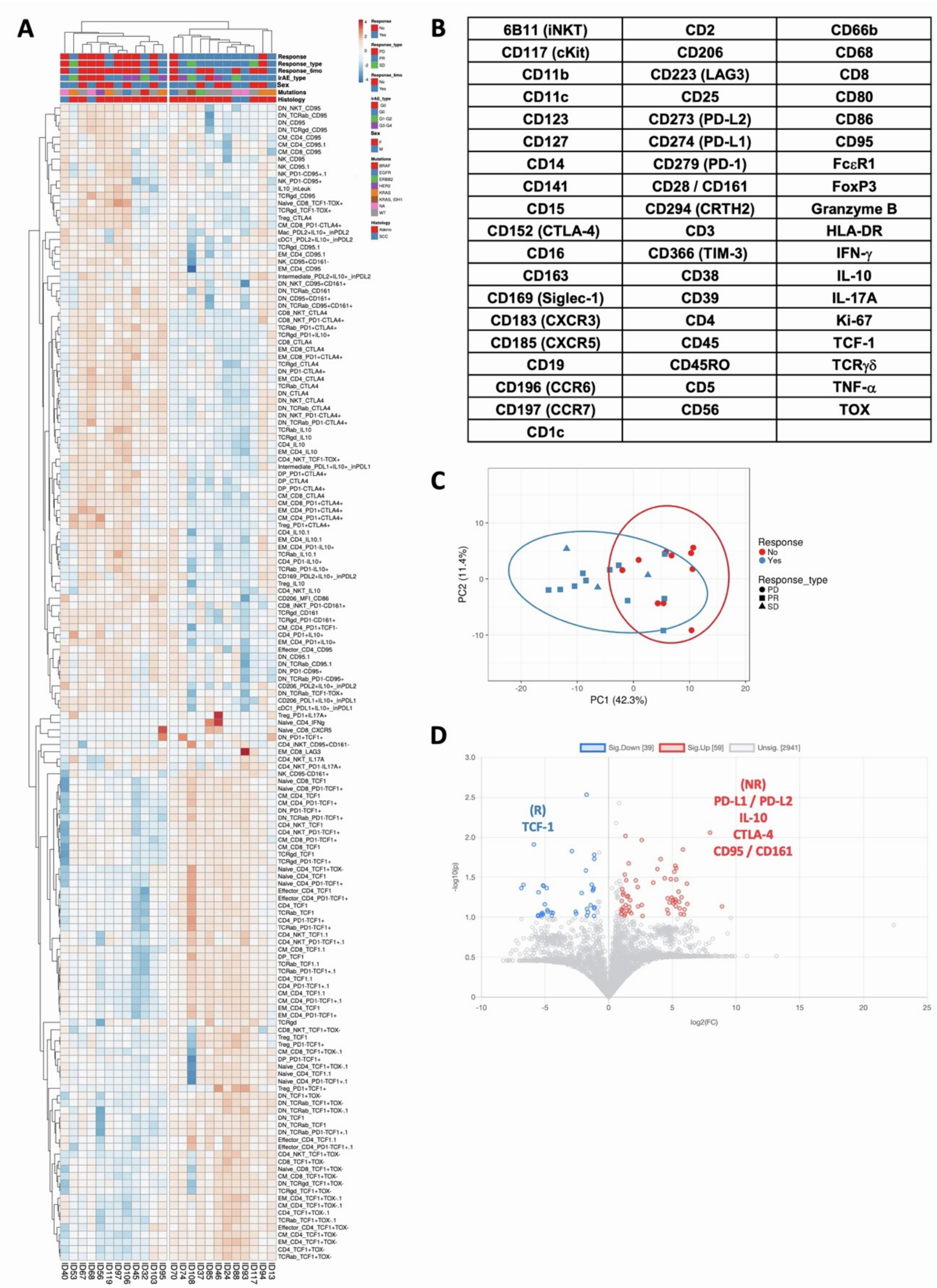

6B11 (iNKT)	CD2	CD66b
CD117 (cKit)	CD206	CD68
CD11b	CD223 (LAG3)	CD8
CD11c	CD25	CD80
CD123	CD273 (PD-L2)	CD86
CD127	CD274 (PD-L1)	CD95
CD14	CD279 (PD-1)	FcεR1
CD141	CD28 / CD161	FoxP3
CD15	CD294 (CRTH2)	Granzyme B
CD152 (CTLA-4)	CD3	HLA-DR
CD16	CD366 (TIM-3)	IFN-γ
CD163	CD38	IL-10
CD169 (Siglec-1)	CD39	IL-17A
CD183 (CXCR3)	CD4	Ki-67
CD185 (CXCR5)	CD45	TCF-1
CD19	CD45RO	TCRγδ
CD196 (CCR6)	CD5	TNF-α
CD197 (CCR7)	CD56	TOX
CD1c		

Figure S3. High dimensional flow cytometry data allows clusterization of responder and non-responder patients to immunotherapy, Related to Figure 3. PBMC from samples collected before starting the treatment were analyzed by high dimensional flow cytometry and patients were identified as either non-responders (red) or responders (blue) at 9th week of treatment (n=10 NR, n=14 R). A total of 3033 leukocyte subpopulations was defined by supervised gating analysis. (**A**) Unsupervised heatmap clusterization of responder and non-responder patients using 150-top subpopulations differentially frequent in these groups (p<0.05). (**B**) All markers evaluated by flow cytometry. (**C**) Principal component analysis (PCA) plots using the same parameters of heatmaps. (**D**) Volcano plot to immune subpopulations significantly downregulated (blue) or upregulated (red) in non-responder patients, including the markers more present in these cell populations.

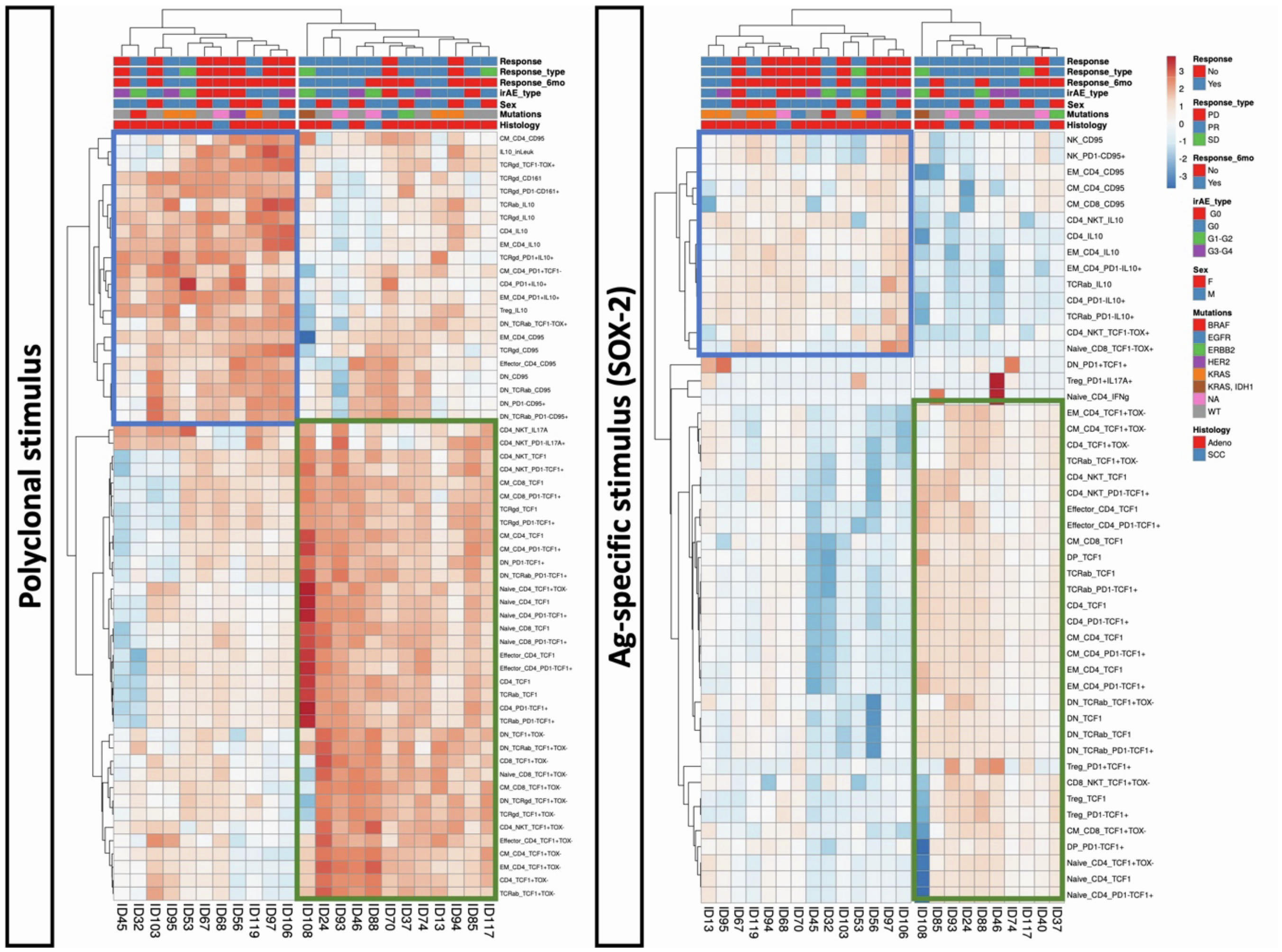

Figure S4. Responder and non-responder patients segregated in two groups based on functional immune cell populations, Related to Figure 4. PBMC from samples collected before starting the treatment were analyzed by high dimensional flow cytometry and patients were identified as either non-responders (red) or responders (blue) at 9th week of treatment (n=9 NR, n=14 R). A total of 980 T lymphocytes subpopulations based on expression of cytokines, transcription factors and functional markers was defined by manual gating analysis. Unsupervised heatmap clusterization of responder and non-responder patients using approximately 50-top subpopulations differentially frequent in these groups ($p<0.05$) after polyclonal stimulation with anti-CD3 and anti-CD28 antibodies or after antigen-specific stimulus with SOX-2 overlapping peptides and anti-CD28 antibody.

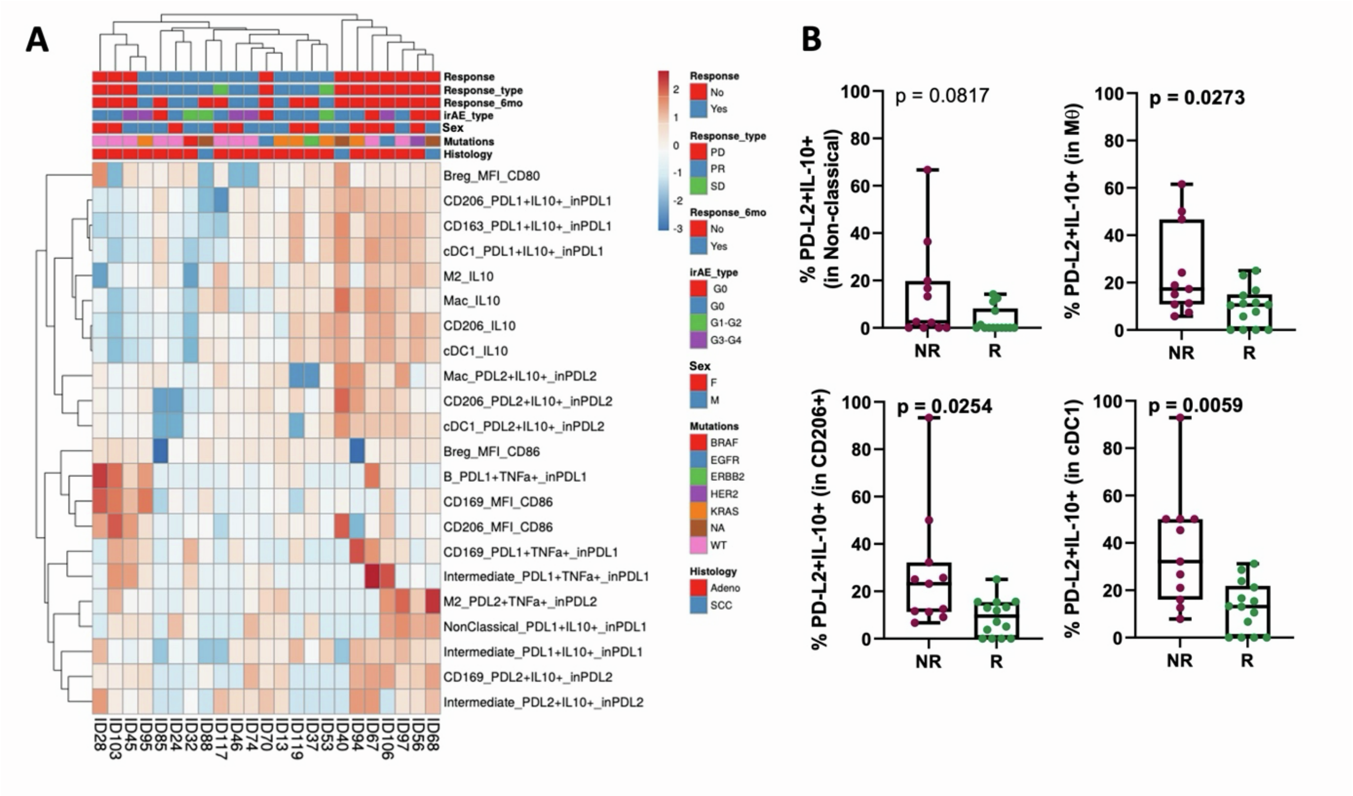

Figure S5. Myeloid cells (CD11b+ cells) expressing PD-L2 and IL-10 are associated with no response to immunotherapy in NSCLC patients, Related to Figure 4. PBMC from samples collected before starting the treatment were analyzed by high dimensional flow cytometry and patients were identified as either non-responders (red and pink) or responders (blue and green) at the 9th week of treatment (n=11 NR, n=14 R). (**A**) Unsupervised heatmap clusterization of responder and non-responder patients using approximately 20-top subpopulations differentially frequent in these groups (p<0.05). (**B**) Frequencies of PD-L2+IL-10+ cells in non-classical monocytes, macrophages, CD206+ cells or cDC1 from responder and non-responder NSCLC patients.

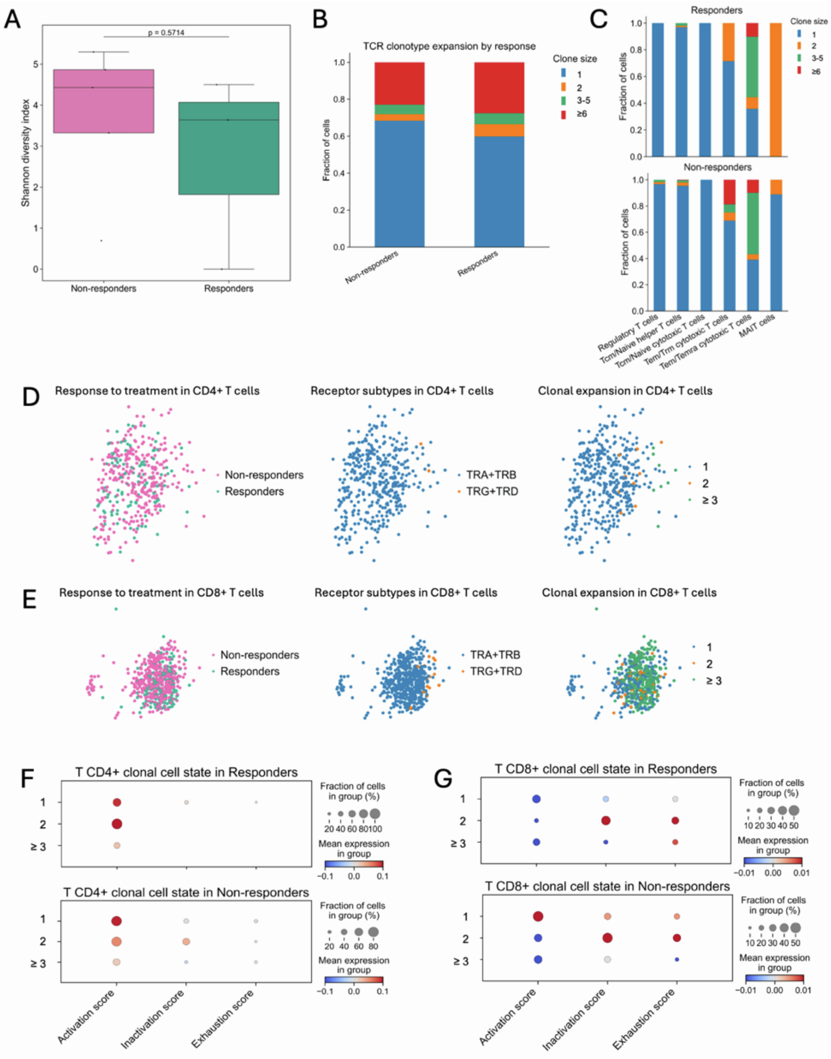

Figure S6. Baseline TCR repertoire diversity and clonal expansion in circulating T cells do not distinguish responders from non-responders, Related to Figure 6. (A) TCR repertoire diversity in responders and non-responders at baseline assessed by the Shannon diversity index, showing no significant difference between groups. Statistical analysis was performed using the Mann–Whitney U test. (B) Global distribution of TCR clonotype expansion by response status, shown as the fraction of circulating T cells belonging to clonotype size categories (1,2,3–5,or ≥6 cells per clonotype), with similar patterns in responders (n= 3; 316 cells) and non-responders (n = 5; 780 cells). (C) Distribution of clonotype sizes across major circulating T cell subsets in responders (top) and non-responders (bottom), revealing expected subset-specific differences but no response-associated shifts. (D) UMAP projections of circulating TCR-expressing CD4^{+} T cells colored by response to treatment (left), TCR receptor subtype (TRA+TRB αβ T cells vs TRG+TRD γδ T cells; middle), and clonal expansion (clonotype size: 1, 2, or ≥3 cells per clonotype; right), showing transcriptional intermixing of responders and non-responders, dominance of αβ T cells, and lack of segregation by clonotype size. (E) UMAP projections of circulating TCR-expressing CD8^{+} T cells colored by the same three features as in panel D, demonstrating greater clonal expansion than CD4^{+} T cells but no response-specific transcriptional clustering. (F) Functional activation, inactivation, and exhaustion gene program scores in CD4^{+} T cells stratified by clonotype size in responders and non-responders, with dot size indicating cell fraction and color indicating mean score, and showing functional states largely independent of clonal expansion. (G) Corresponding functional gene program scores in CD8^{+} T cells, showing higher exhaustion-associated scores in expanded clonotypes in responders compared with non-responders. Activation, inactivation, and exhaustion scores were calculated as module scores using the following gene sets: activation (TCF7, CD69, LAMP1, CXCR3), inactivation (FAS, KLRB1, IL10, PDCD1LG2), and exhaustion (CTLA4, LAG3, PDCD1, TIGIT).

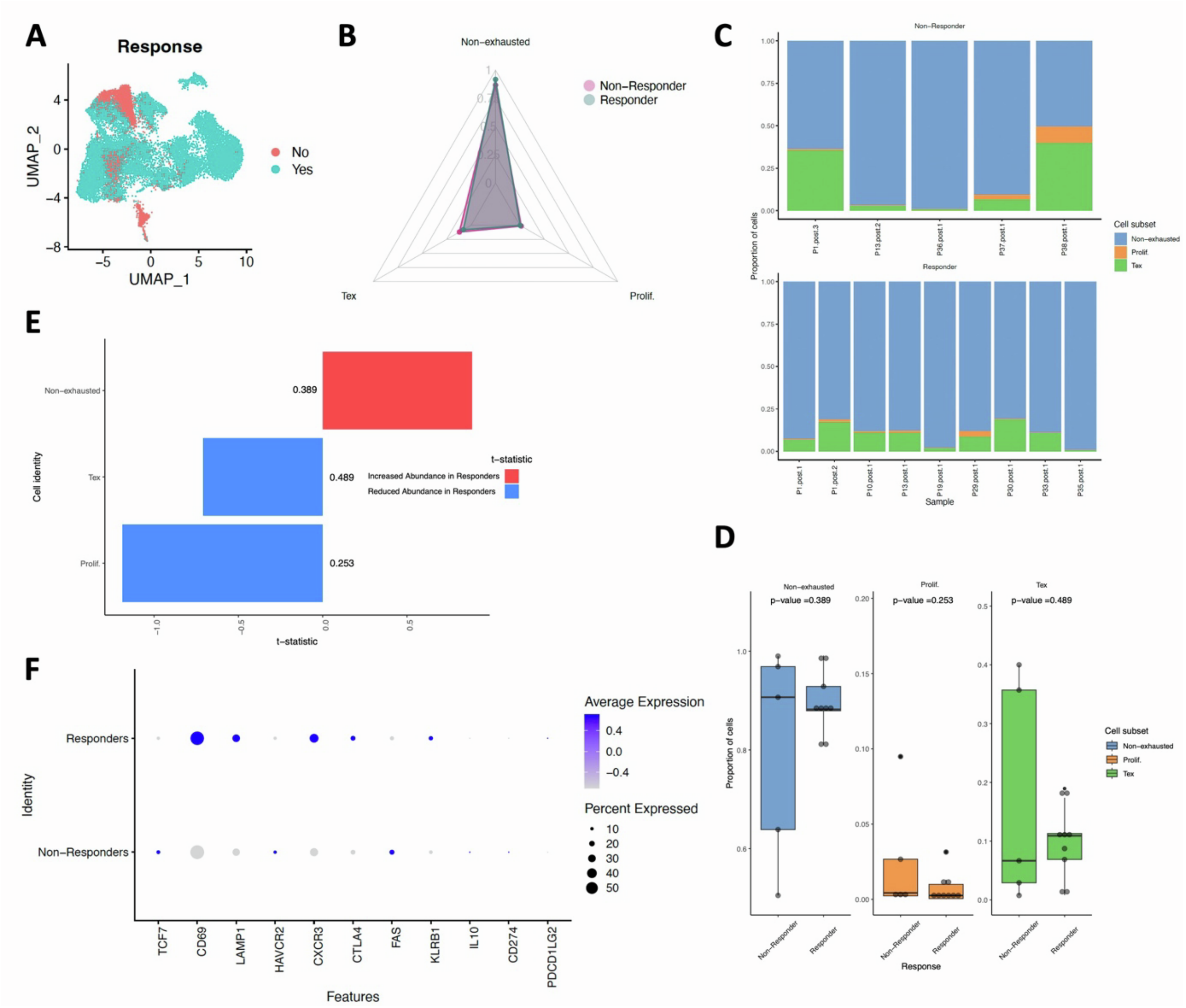

Figure S7. Single-Cell analysis of CD8 T lymphocyte subpopulation differences in response to immunotherapy for NSCLC patients from the GEO database (GSE179994), Related to Figure 7. (**A**) UMAP plot reveals the distribution of CD8 T lymphocytes from responders (in blue) and non-responders (in red) (n=5 NR, n=9 R). (**B**) Radar plot provides an overview of the proportions of distinct CD8 T lymphocyte subpopulations in responders (in blue) and non-responders (in red) from all samples. (**C**) The stacked barplot shows the heterogeneity in the proportions of CD8 T lymphocyte subpopulations across all samples in both groups. (**D-E**) The boxplots and the horizontal barplot present the results of the differential abundance test. Boxplots depict the frequencies of each cell population, along with their respective p-values comparing responders and non-responders. The horizontal barplot shows the log2 fold change in abundance, with red indicating increased abundance in responders and blue indicating reduced abundance in responders. (**F**) The dotplot illustrates the expression of markers of CD8 T lymphocyte subpopulations. Dot size corresponds to the percentage of cells expressing each gene marker in each group, and colors represent the average expression level. The CD8 T lymphocyte subpopulations are identified as CD8 Non-exhausted: Activated effector cells, CD8 Prolif: Proliferative cells, CD8 Tex: Terminally exhausted cells.

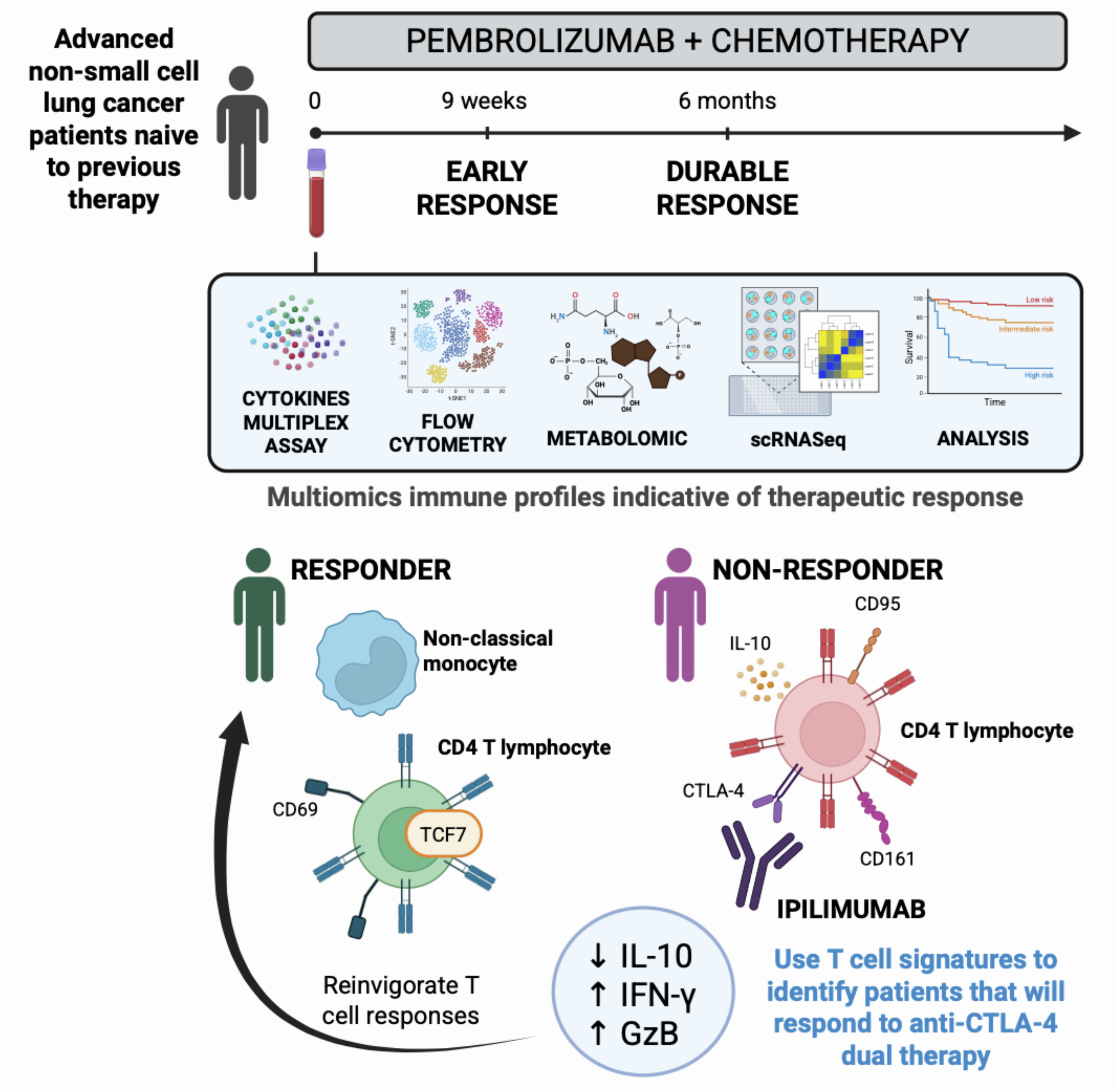

Figure S8. Immunological signatures in NSCLC patients suggesting personalized immunotherapy, Related to Discussion session. This study enrolled 33 stage IV NSCLC patients undergoing anti-PD1 therapy with chemotherapy and suggests that distinct immune profiles may predict treatment outcomes. Responders exhibited higher levels of activated CD4+CD69+ T cells and non-classical monocytes, while non-responders presented an immunosuppressive profile with increased frequencies of CTLA-4, CD161, CD95, and IL-10 expressing T cells. Pre-existing activated CD4+ T cells correlated with extended progression-free survival. Combining PD-1 and CTLA-4 blockade reinvigorated anti-tumor responses from CD4 and CD8 T cells. This investigation emphasizes the pivotal role of CTLA-4-expressing T cells in NSCLC, fostering an immunosuppressive milieu linked to treatment resistance.

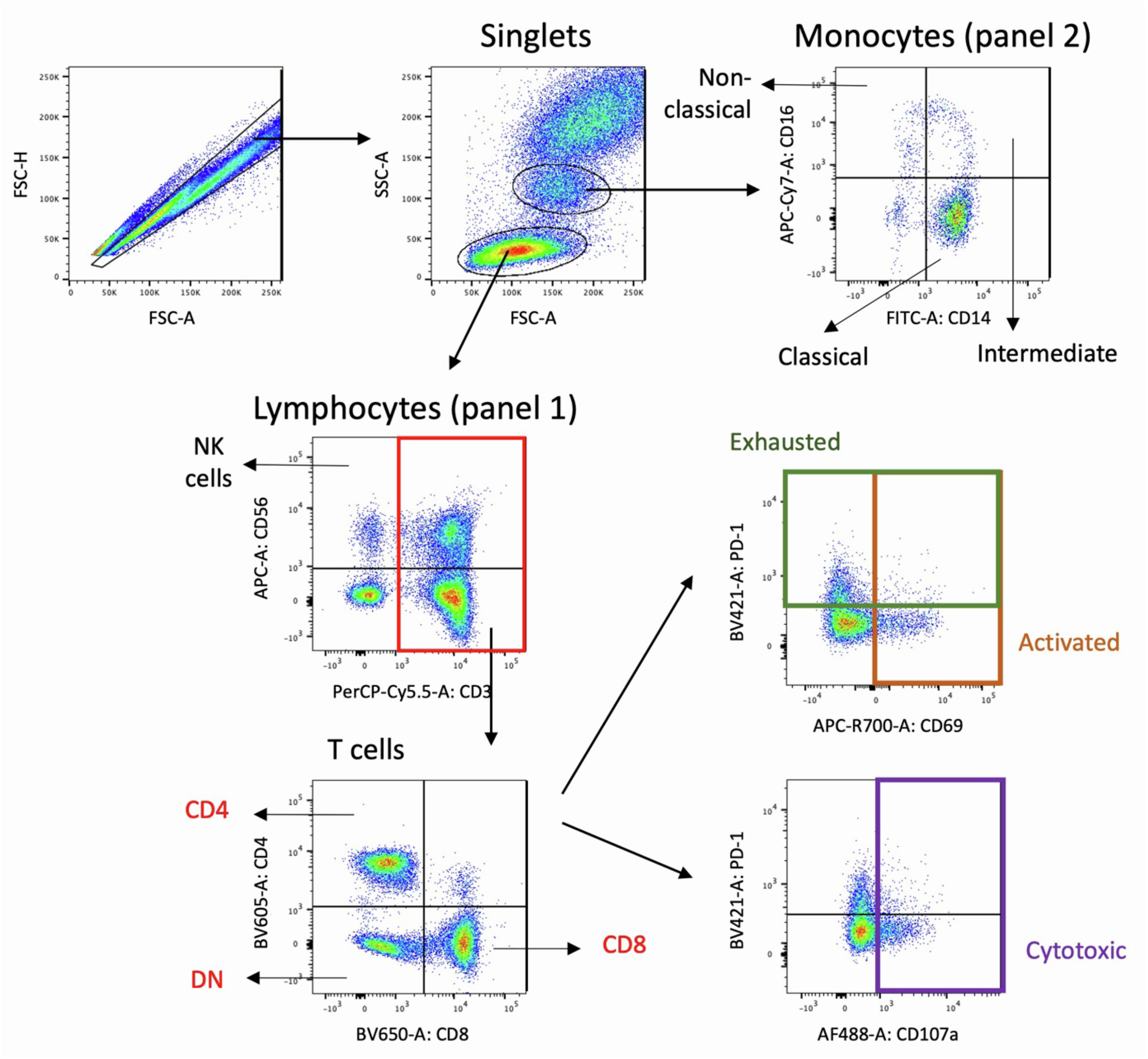

Figure S9. Analysis strategy for flow cytometry data from whole blood samples, Related to Flow cytometry STAR Methods.

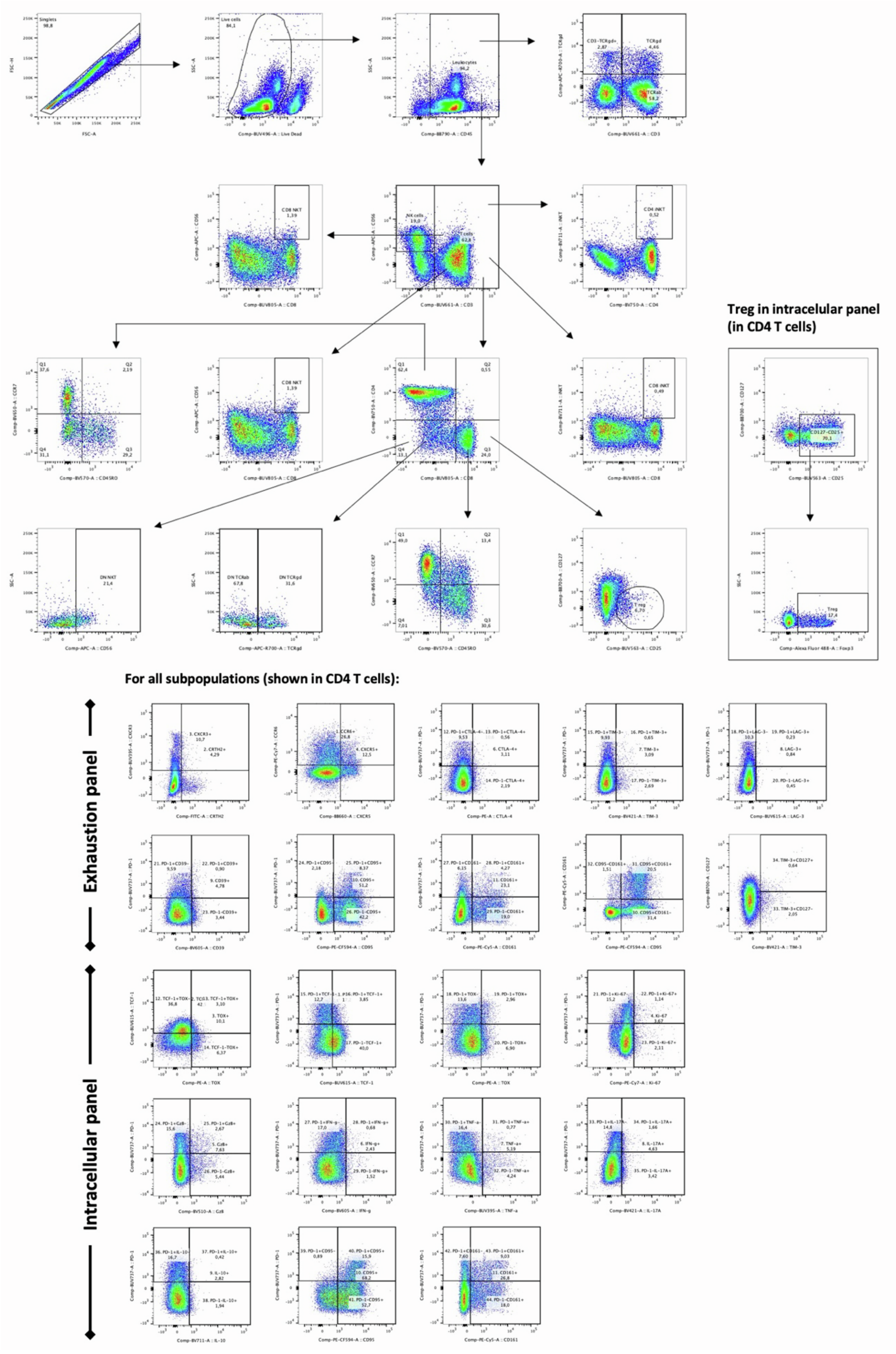

Figure S10. Analysis strategy for flow cytometry data from PBMC samples – NK cells and T lymphocytes populations, Related to Flow cytometry STAR Methods.

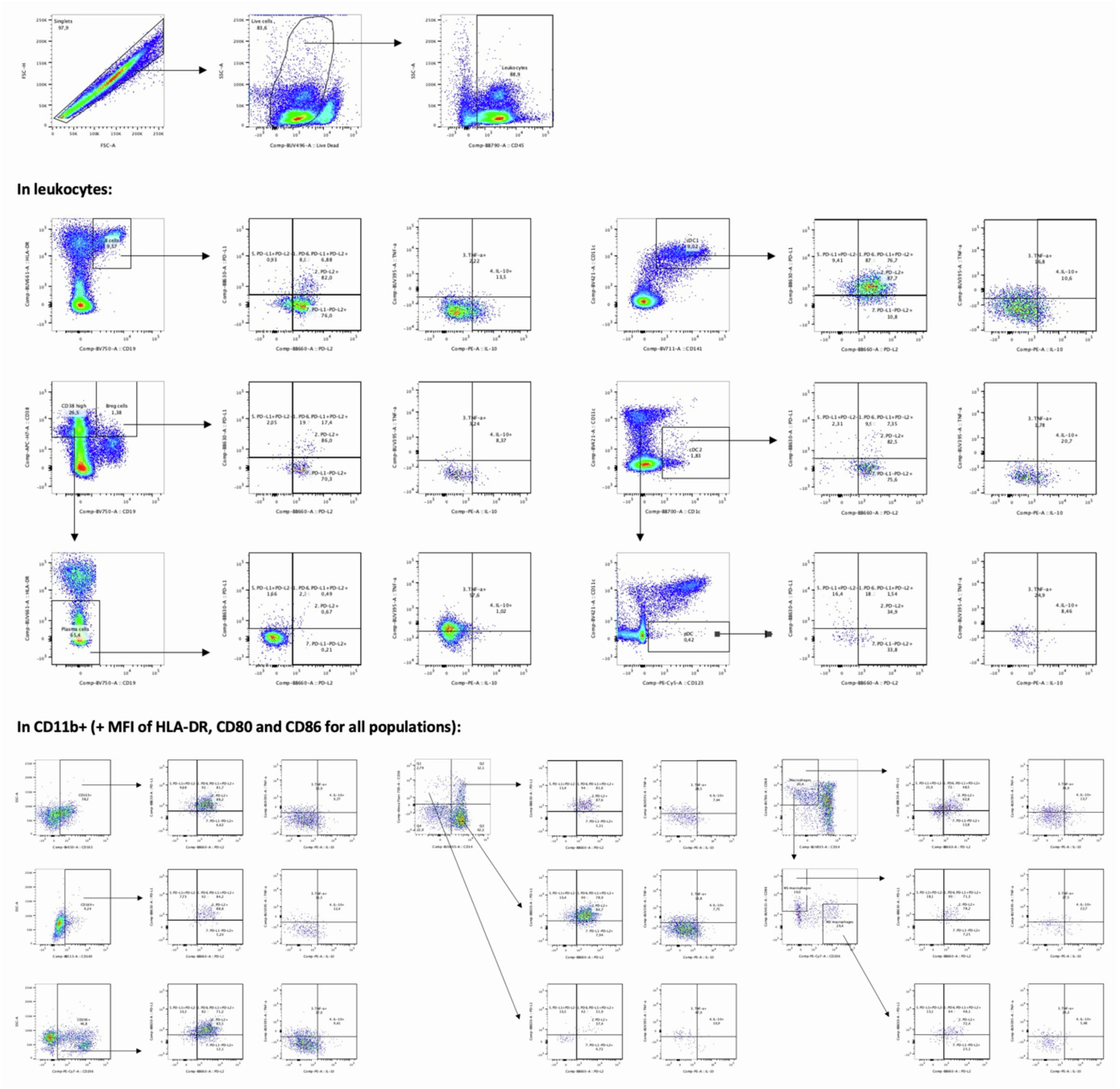

Figure S11. Analysis strategy for flow cytometry data from PBMC samples – B lymphocytes and myeloid populations, Related to Flow cytometry STAR Methods.

Table S1: Patient demographics and clinical characteristics, Related to Cohort characterization and Figure 1.

	Non-responders (n=11) % (N)	Responders (n=22) % (N)	p-value
Type of response			-
Disease Progression	33.3 (11)		
Stable disease		24.2 (8)	
Partial response		42.5 (14)	
Age<br>Median (range)	62.5 (26.2 - 79.4)	69 (48.8 – 80.8)	0.0492
Sex			0.4813
Female	54.5 (6)	45.5 (10)	
Male	45.5 (5)	54.5 (12)	
Site of metastasis			0.3618
Bone	66.7 (8)	33.3 (7)	
CNS	8.3 (1)	23.8 (5)	
Lung	25 (3)	23.8 (5)	
Other	33.3 (4)	47.6 (10)	
Histology			1.000
Adenocarcinoma	81.8 (9)	81.8 (18)	
Squamous cell carcinoma	18.2 (2)	18.2 (4)	
Driver mutation			0.2880
KRAS	9.1 (1)	28 (6)	
BRAF	0	4.5 (1)	
EGFR (*)	18.2 (2)	4.5 (1)	
ERBB2	0	9 (2)	
WT	54.5 (6)	36 (8)	
NA	18.2 (2)	18 (4)	
PD-L1 expression			0.2798
0	63.6 (7)	33.3 (7)	
1-5%	27.3 (3)	28 (6)	
> 5%	9.1 (1)	38 (8)	
NA	0	(1)	
Treatment regimens			1.000
Pembro+Carboplatin+Pemetrexed	81.8 (9)	81.8 (18)	
Pembro+Carboplatin+Paclitaxel	18.2 (2)	18.2 (4)	
Follow-up (months)<br>Median (range)	7.5 (1.5 – 29.8)	20.2 (8.5 – 36.5)	0.0117
Overall survival (months)<br>Median	7.8	14.3	0.0227

CNS: central nervous system.

(*) TKI-insensitive mutations.

Table S2: Discriminant metabolites of treatment response at 9 weeks identified in metabolomics analysis, Related to Figure 1.

m/z	RT (min)	p-value*	of change	Identification (adduct, m/z error in ppm)	Type of ID
135.0319	1.5	0.076	-17.7	Hypoxantinhe ($[M-H]^-$, 5 ppm)	*in-house* library (RT & MS/MS)
151.0266	1.8	0.021	-30.1	Xanthine ($[M-H]^-$, 3 ppm)[a]	*in-house* library (RT & MS/MS)
288.2156	6.8	0.077	51.1	Octanoylcarnitine ($[M+H]^+$, 5 ppm)	MS/MS (Class specific fragments)
308.1005	1.5	0.026	-22.0	N-Acetylneuraminate ($[M-H]^-$, 6 ppm)	*in-house* library (RT & MS/MS)
314.2325	7.5	0.094	52.6	Decenoylcarnitine ($[M+H]^+$, 0 ppm)	MS/MS (Class specific fragments)
316.2475	7.9	0.094	61.4	Decanoylcarnitine ($[M+H]^+$, 2 ppm)	MS/MS (Class specific fragments)
346.0584	2.0	0.045	-24.7	Adenosine Monophosphate ($[M-H]^-$, 3 ppm)	*in-house* library (RT & MS/MS)
391.2901	11.3	0.041	64.8	Ursodeoxycholate/ Deoxycholate ($[M-H]^-$, 12 ppm)[b]	*in-house* library (RT & MS/MS)
397.2067	12.4	0.054	-29.4	Pregnenolone sulfate ($[M-H]^-$, 3 ppm)	MS/MS (External library match)
498.2923	11.5	0.015	-47.9	Taurodeoxycholate/ Tauroursodeoxycholate/ Taurochenodeoxycholate ($[M-H]^-$, 6 ppm)	MS/MS (Class specific fragments)
504.3128	11.6	0.034	34.5	LPC(18:2) ($[M-CH_3]^-$, 8 ppm)	MS/MS (Characteristic fragmentation: class and fatty acyl composition)

* Mann-Whitney-Wilcoxon test for data without normal distribution, and the Student's t test for data with normal distribution was applied to distinguish responders from non-responders at time 0.

[a] oxypurinol is not available at our in-house library and presents the same MS/MS pattern as xanthine.

[b] chenodeoxycholic acid is not available at our in-house library and presents the same MS/MS pattern as deoxycholate and ursodeoxycholate.

Table S3. Genes expressed in CD4 and CD8 subpopulations found in scRNASeq analysis of the GEO database (GSE179994), Related to Figure 7.

Population	Main expressed genes
CD4-Naive	CCR7
CD4-Tcm	ANXA1, LMNA, MYADM, RGCC
CD4-Tem	GZMA, CCL5, GZMK
CD4-CD69	FOS, FOSB, DUSP1, CD69
CD4-ISG15	IFI27, ISG15, IFI6, LY6E
CD4-RPL	RPS29, RPL41, RPS27, TCF7
CD4-Th1-like	CXLCL13, TOX, PDCD1, IFNG
CD4-Treg	LAYN, CCR8, FOXP3
CD4-Prolif	MKI67, STMN1, TYMS, TUBA1B, TUBB, UBA52, CRIP1, TNFRSF9, CD69
CD4-XCL1	XCL1
CD8-Non-exhausted	GPR183, IL7R, LMNA, ANXA1, CCR7, TUBA4A, CD28, CXCR4, HSPA1A, DNAJA1, HSPA1B, MYADM, FKBP5, TXNIP, GZMA, CXCR6, FOS, CCL4, CCL4L2, FOSB, CD69, GZMK
CD8-Tex	TIGIT, HAVCR2, ITGAE
CD8-Prolif	MKI67, STMN1, ENTPD1

Table S4. Patient individual demographics and clinical characteristics, Related to Study participant details STAR Methods.

Patient ID	Percent PD-L1	Histology	Sex	Age (years)	Mutations	Early Response (9 weeks)	Response type (9 weeks)*	Durable Response (6 months)	irAE**
ID13	1	Adeno	M	64.8	KRAS	Yes	PR	Yes	G0
ID21	0	Adeno	M	48.8	WT	Yes	PR	Yes	G0
ID24	60	Adeno	F	73.0	WT	Yes	PR	Yes	G0
ID28	5	Adeno	F	69.1	WT	No	PD	No	G0
ID32	1	Adeno	M	78.8	BRAF	Yes	PR	Yes	G1-G2
ID37	5	Adeno	F	69.9	ERBB2	Yes	PR	No	G0
ID40	0	SCC	M	61.5	NA	No	PD	No	G0
ID45	0	Adeno	M	26.2	WT	No	PD	No	G3-G4
ID46	0	Adeno	F	68.5	WT	Yes	PR	Yes	G3-G4
ID53	0	Adeno	M	74.2	KRAS	Yes	SD	Yes	G1-G2
ID56	0	Adeno	F	79.4	HER2	No	PD	No	G0
ID58	1-5	Adeno	F	73.8	EGFR	Yes	SD	Yes	G0
ID67	0	Adeno	F	59.6	WT	No	PD	No	G0
ID68	70	SCC	M	66.4	NA	No	PD	No	G0
ID70	5	Adeno	M	52.8	EGFR	No	PD	No	G0
ID74	60	Adeno	M	68.7	WT	Yes	PR	Yes	G3-G4
ID77	NA	SCC	M	65.6	NA	Yes	SD	Yes	G0
ID85	0	Adeno	M	63.9	WT	Yes	PR	No	G0
ID88	10	SCC	M	78.6	NA	Yes	PR	No	G1-G2
ID93	0	SCC	M	70.8	NA	Yes	PR	Yes	G0
ID94	0	Adeno	F	62.5	KRAS	No	PD	No	G0
ID95	40	Adeno	M	75.7	KRAS	Yes	PR	Yes	G3-G4

ID97	0	Adeno	M	65.6	WT	No	PD	No	G0
ID99	0	Adeno	F	79.3	WT	Yes	SD	Yes	G0
ID103	0	Adeno	F	57.0	WT	No	PD	No	G0
ID106	1	Adeno	F	74.9	EGFR	No	PD	No	G3-G4
ID108	80	Adeno	M	67.6	KRAS, IDH1	Yes	SD	Yes	G0
ID111	< 1	Adeno	M	80.8	ERBB2	Yes	SD	Yes	G1-G2
ID117	80	Adeno	F	67.2	WT	Yes	SD	No	G0
ID118	30	Adeno	F	55.9	WT	Yes	PR	Yes	G0
ID119	0	Adeno	F	63.9	KRAS	Yes	PR	No	G0
ID120	30	Adeno	F	69.3	KRAS, NRAS	Yes	PR	Yes	G1-G2
ID134	1	SCC	F	68.0	NA	Yes	SD	No	G0

* PD = Progressive Disease, PR = Partial Response, SD = Stable Disease
** irAE = immune related adverse events as classified by grades 0, 1-2 or 3-4.

Table S5. Antibodies used for flow cytometry, Related to Flow cytometry STAR Methods.

Marker	Clone	Fluorochrome
Whole blood labeling		
CD107a	H4A3	Alexa Fluor 488
CD11b	D12	BV650
CD11c	B-ly6	PerCP-Cy5.5
CD14	M5E2	FITC
CD152 (CTLA-4)	BNI3	PE
CD16	3G8	APC-Cy7
CD19	HIB19	PE
CD273 (PD-L2)	MIH18	APC-R700
CD274 ()	MIH1	BV421
CD279 (PD-1)	MIH4	BV421
CD3	UCHT1	PerCP-Cy5.5
CD38	HB7	PE-Cy7
CD4	SK3	BV605
CD56	B159	APC
CD69	FN50	APC-R700
CD8	RPA-T8	BV650
HLA-DR	G46-6	APC
PBMC labeling		
6B11 (iNKT)	6B11	BV711
CD117 (cKit)	YB5.B8	PE-CF594
CD11b	ICRF44	BV570
CD11c	B-ly6	BV421
CD123	9F5	PE-Cy5
CD127	HIL-7R-M21	BB700
CD14	M5E2	BUV805
CD141	1A4	BV711
CD15	W6D3	BV510
CD152 (CTLA-4)	BNI3	PE
CD16	3G8	Alexa Fluor 700
CD161	DX12	PE-Cy5
CD163	GHI/61	BV650
CD169 (Siglec-1)	7-239	BB515
CD183 (CXCR3)	1C6/CXCR3	BUV395
CD185 (CXCR5)	RF8B2	BB660
CD19	SJ25C1	BV510
CD19	SJ25C1	BV750
CD19	SJ25C1	BV786
CD196 (CCR6)	11A9	PE-Cy7
CD197 (CCR7)	2-L1-A	BV650
CD1c	F10/21A3	BB700
CD2	RPA-2.10	BUV737
CD206	19.2	PE-Cy7
CD223 (LAG-3)	T47-530	BUV615
CD25	2A3	BUV563
CD273 (PD-L2)	MIH18	BB660

CD274 ()	MIH1	BB630
CD279 (PD-1)	EH12.1	BUV737
CD28	CD28.2	PE-Cy5
CD294 (CRTH2)	BM16	FITC
CD3	UCHT1	BUV661
CD366 (TIM-3)	7D3	BV421
CD38	HB7	APC-H7
CD39	TU66	BV605
CD4	SK3	BV750
CD45	HI30	BB790
CD45RO	UCHL1	BV570
CD5	UCHT2	BV786
CD56	B159	APC
CD66b	G10F5	Alexa Fluor 647
CD68	Y1/82A	BV786
CD73	AD2	BV510
CD8	SK1	BUV805
CD80	L307.4	BB755
CD86	2331 (FUN-1)	BUV615
CD95	l307.4	PE-CF594
Eomes	X4-83	PE
FCεR1	AER-37	BV605
Foxp3	259D/C7	Alexa Fluor 488
Granzyme B	GB11	BV510
HLA-DR	G46-6	BUV661
IFN-γ	B27	BV605
IL-10	JES3-9D7	BV711
IL-10	JES3-9D7	PE
IL-17A	N49-653	BV421
Ki-67	B56	PE-Cy7
TCF-1/TCF-7	S33-966	BUV615
TCRγδ	11F2	APC-R700
TNF-α	MAb11	BUV395
TOX	TXRX10	PE
